# Supplementary material for: Automatic structure-based NMR methyl resonance assignment in large proteins
Source: Nat Commun. 2019 Oct 29;10:4922. doi: 10.1038/s41467-019-12837-8 (PMC6820720; doi:10.1038/s41467-019-12837-8)
Supplement: Supplementary file 1 — Supplementary Information [file 41467_2019_12837_MOESM1_ESM.pdf]

Supplementary Information

Automatic structure-based methyl NMR resonance assignment in large proteins

Iva Pritišanac et al.

Contents

Supplementary Fig. 1 Parametrization of the distance cutoff d_{cut} and the probability of expected methyl-methyl NOE contacts, p_{NOE}.

Supplementary Fig. 2 Accuracy of the methyl assignments obtained for the different values of NOE probabilities over a range of NOE distance cutoffs, d_{cut}.

Supplementary Fig. 3 Determination of the optimal number of individual assignment runs for automatic methyl resonance assignment with MethylFLYA.

Supplementary Table 1 Methyl resonance assignments by MethylFLYA (labeled FY), MAGMA (labeled MG), MAP-XSII (labeled MP), FLAMEnGO2.0 (labeled FL), and MAGIC (labeled MA, where applicable).

Supplementary Fig. 4 Sources of errors in the automatic methyl resonance assignments generated by MethylFLYA.

Supplementary Table 2 Summary of errors in the automatic methyl resonance assignments generated with MethylFLYA.

Supplementary Fig. 5 Parameter optimization for automatic NOESY peak picking with CYPICK.

Supplementary Fig. 6 2D $^{13}C(\omega_1)$-$^{13}C(\omega_2)$ projections of 3D CCH NOESY (ATCase, HSP90) or 4D HCCH NOESY spectra (EIN).

Supplementary Table 3 MethylFLYA computation times (h) for different combinations of input NMR data, as in Fig. 3.

Supplementary Table 4 Results of the CYPICK application to the 3D CCH NOESY (ATCase, HSP90) and 4D HCCH NOESY (EIN) spectra.

Supplementary Table 5 Description of the input data for the MethylFLYA runs using minimal data input.

Supplementary Fig. 7 Summary of the results of methyl resonance assignment of EIN using MethylFLYA with no knowledge of the reference ^{1}H-^{13}C resonance positions and methyl residue types, as detailed in Supplementary Table 5.

Supplementary Fig. 8 Correlations between the measured Alanine methyl chemical shifts for EIN and those predicted with SHIFTX2 based on the crystal structure (PDB ID: 1EZA).

Supplementary Fig. 9 MethylFLYA performance on different input structures for three enzymes in the benchmark

Supplementary Table 6 Results of the MAGIC protocol runs on ATCase, EIN, and HSP90, using, where applicable, both filtered and unfiltered NOESY peak lists.

Supplementary Fig. 10 Performance of the MAGIC protocol as summarized in Supplementary Table 6.

Supplementary Fig. 11 Intersection of assignments generated with different automatic methyl assignment protocols.

Supplementary Methods

Supplementary References

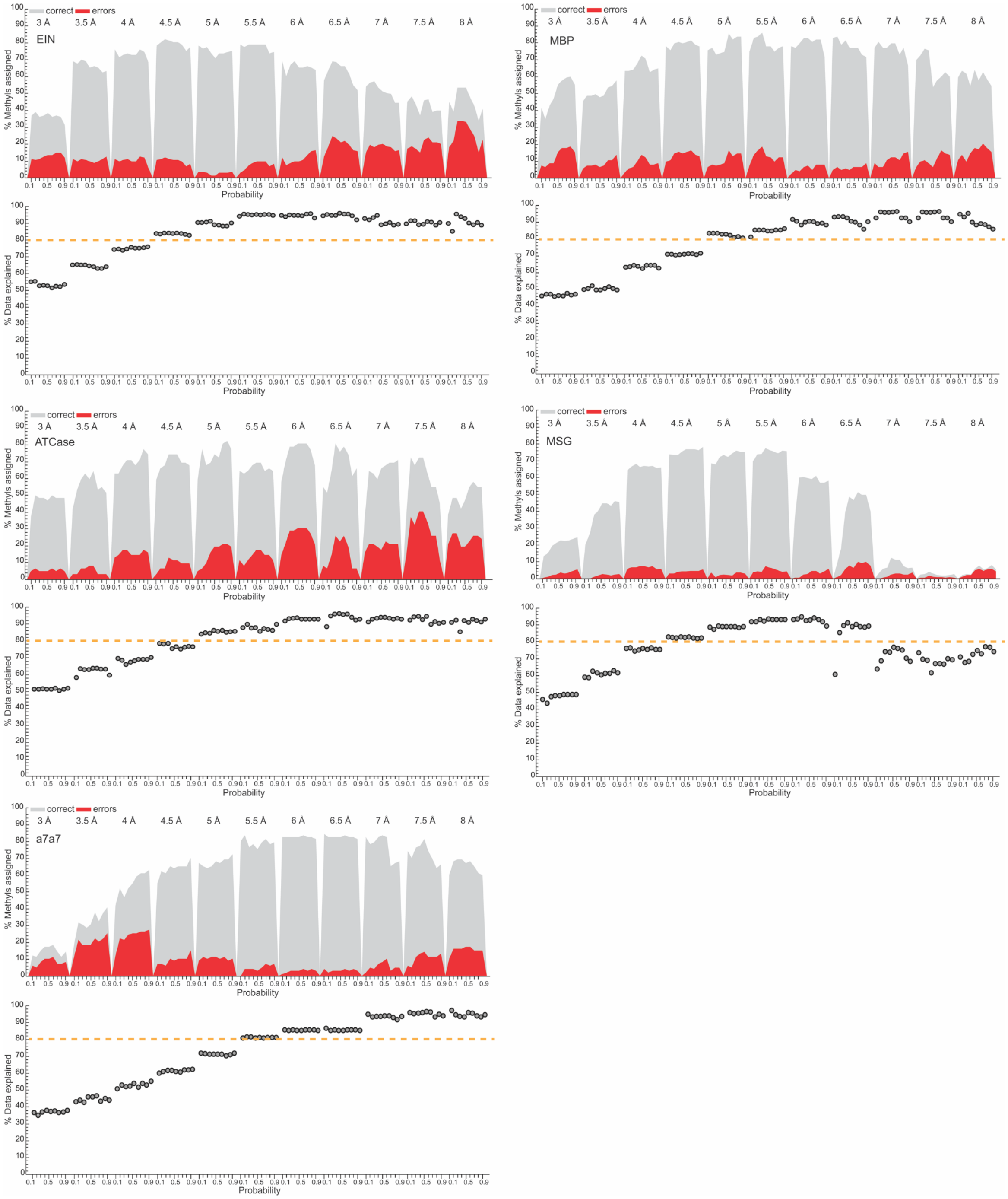

Supplementary Fig. 1 Parametrization of the distance cutoff d_{cut} and the probability of expected methyl-methyl NOE contacts, p_{NOE}. For each protein, the upper panel shows the percentage of correct (*grey*) and erroneous (*red*) strong (i.e. confident) assignments for given d_{cut} and p_{NOE} values. Assignment percentages are relative to the number of reference assignments. For each protein, the lower panels show for each protein the percentage of explained experimental NOESY peaks. The distances at which ~80–85% of the data are explained generally led to the most reliable assignments. For the largest dataset. MSG, the algorithmic performance significantly deteriorates for d_{cut} > 6.5 Å (equivalent to >11.5 Å C–C distance), resulting in an unreliable assignment. Note that such large distance cutoffs are unrealistic in practice and unlikely to be generally required.

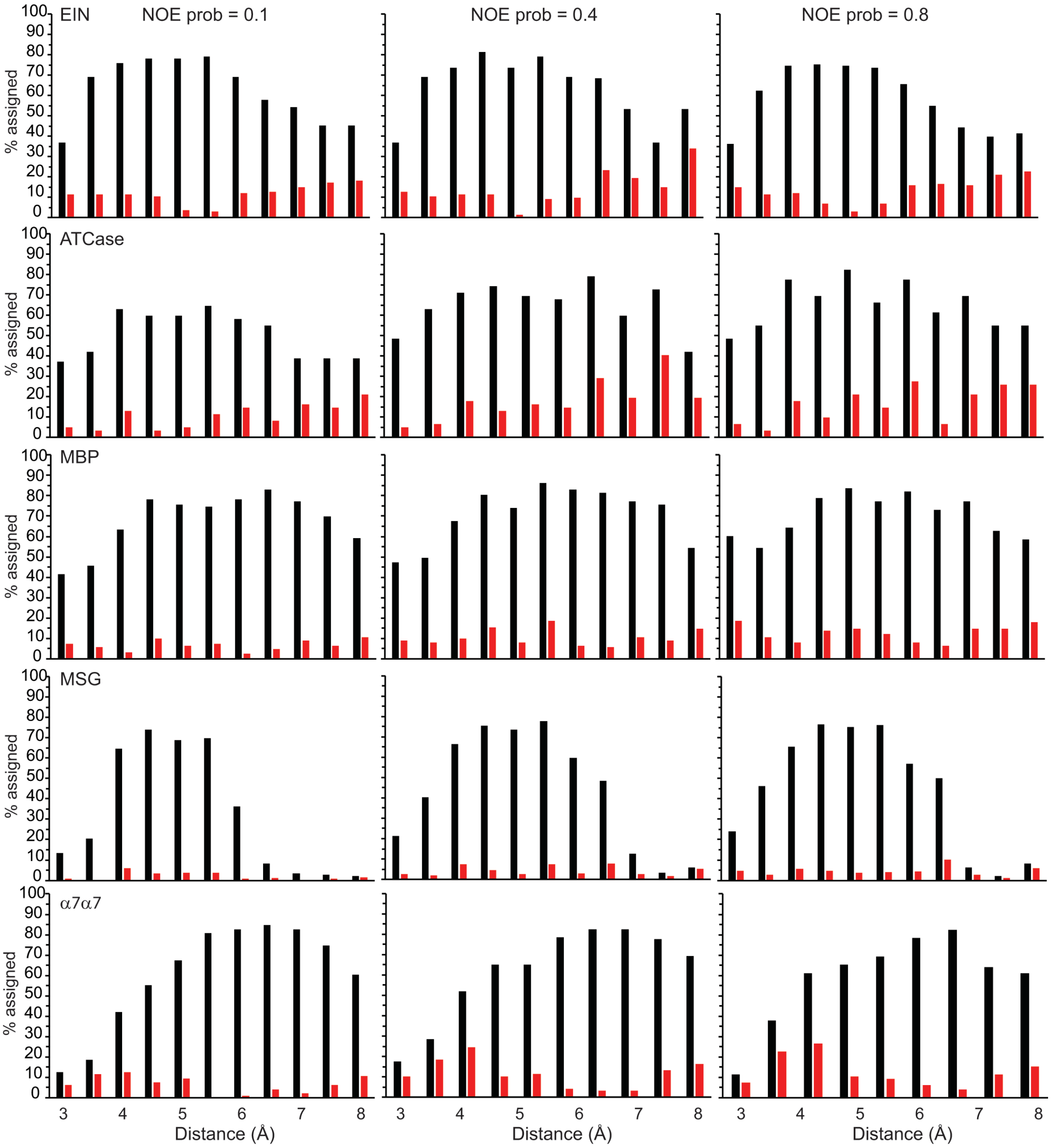

Supplementary Fig. 2 Accuracy of the methyl assignments obtained for the different values of NOE probabilities over a range of NOE distance cutoffs, d_{cut}. The percentage of accurately (*black*) and erroneously (*red*) assigned methyl groups is shown for the NOE probability values of 0.1, 0.4, and 0.8 in the first, second, and third column, respectively. Note that these results are a subset of the results presented in Supplementary Fig. 1.

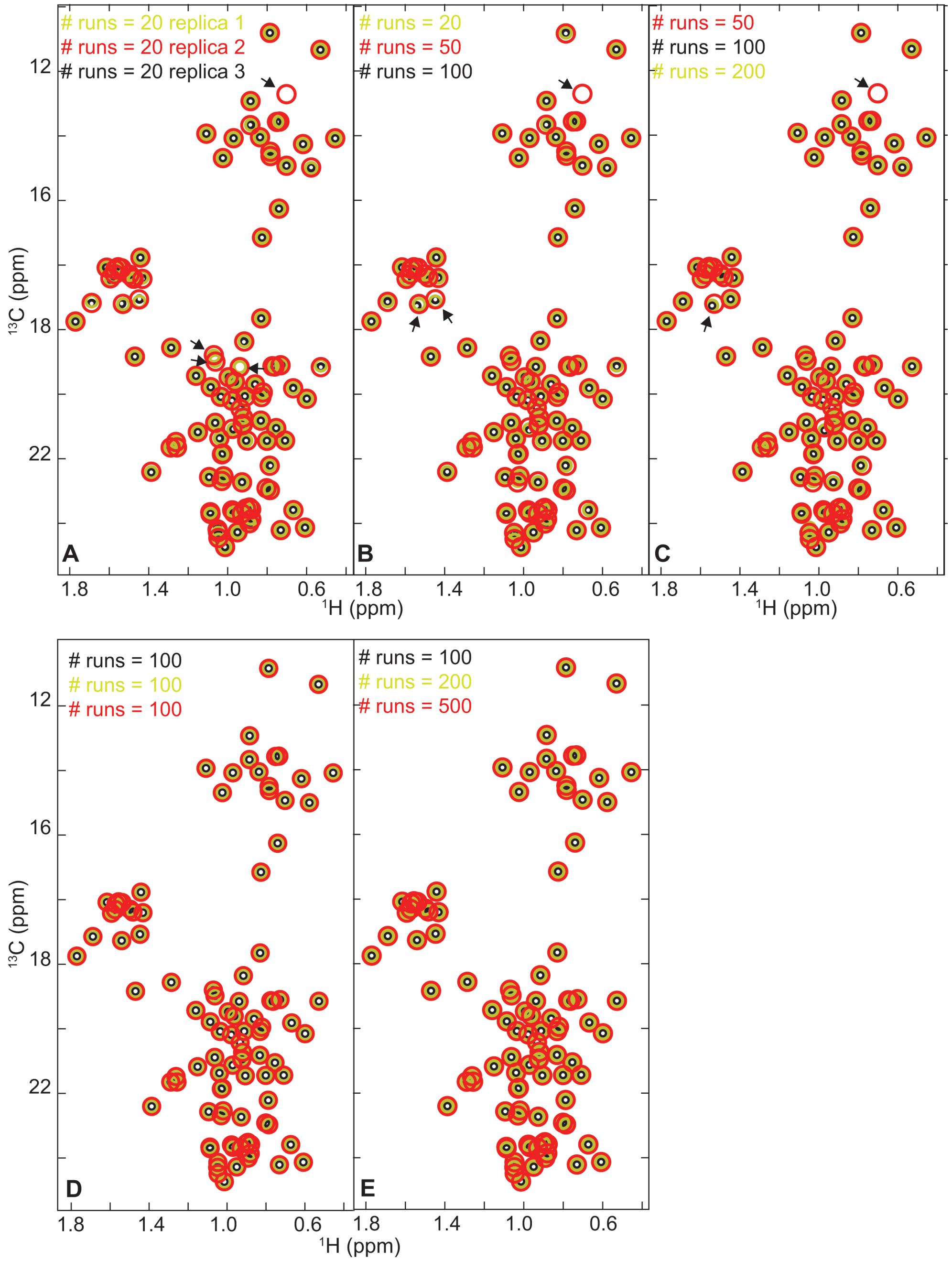

Supplementary Fig. 3 Determination of the optimal number of individual assignment runs for automatic methyl resonance assignment with MethylFLYA. Positions of the "strong" (i.e. confident) MethylFLYA-derived methyl assignments for EIN are shown in ^{1}H-^{13}C correlation plots as circles with different colors and increasing diameters. **A** Running 20 parallel assignment calculations in three replicates, each from a different random starting point, shows some differences in the derived strong assignments between the replicates (arrows). **B, C** Increasing the number of calculations to 50 shows that the differences persist when compared to the higher number of parallel calculations (100 or 200). **D** Running 100 parallel MethylFLYA calculations is sufficient for the reproducibility of strong assignments. **E** A further increase in the number of parallel calculations (e.g. 200, 500) results in sets of strong assignments that are consistent with the set of 100 parallel calculations.

Supplementary Table 1. Methyl resonance assignments by MethylFLYA (labeled FY), MAGMA[1] (labelled MG), MAP-XSII[2] (labelled MP), FLAMEnGO2.0[3] (labeled FL), and MAGIC[4] (labeled MA, where applicable). The methyl groups with reference assignment are listed, with the ^{1}H and ^{13}C chemical shift values given in ppm in columns '^{1}H' and '^{13}C'. Agreement or disagreement of the confident assignments by the different algorithms with the reference assignment is indicated by '=' (agreement) or '!' (disagreement) signs. The first sign is for ^{1}H, the second for ^{13}C. Missing, non-confident, or ambiguous assignments are left blank. In the case of MethylFLYA, the weak (non-confident) assignments are additionally included in parentheses. Filtered peak lists were used.

EIN

Methyl	^{1}H	^{13}C	FY	MG	MP	FL	MA
2 ILE CD1	0.709	14.560					
5 ILE CD1	0.986	13.190			!!		
6 LEU CD1	0.755	23.060	==	==			==
6 LEU CD2	0.891	25.480	==	==			==
7 ALA CB1	1.342	21.370					
11 ILE CD1	0.834	14.030	==	==	==		==
12 ALA CB1	1.258	23.660	==	==	==		==
16 ALA CB1	1.287	20.550	==	==	==		==
17 LEU CD1	0.884	25.850	==		!!		
17 LEU CD2	1.025	23.840	==		!!		
18 LEU CD1	0.978	25.640	==	==	!!		
18 LEU CD2	0.800	23.450	==	==			==
19 LEU CD1	0.963	25.500	(==)		!!		
19 LEU CD2	0.890	23.980	(==)		!!		
24 ILE CD1	0.970	14.070	==	==	==		==
25 VAL CG1	0.878	20.870					
25 VAL CG2	0.885	20.440			!!		
26 ILE CD1	0.531	11.330	==	==	==		==
31 ILE CD1	0.697	14.940	==	==	==		==
33 ALA CB1	1.573	18.340	==	==			==
36 VAL CG1	1.087	21.790	==	==			==
36 VAL CG2	1.255	23.440	==	==	==		==
40 VAL CG1	1.161	21.440	==	==	==		==
40 VAL CG2	1.294	23.690	==	==	==		==
44 LEU CD1	0.669	21.810	==	==			==
44 LEU CD2	0.927	25.730	==	==			==
48 ALA CB1	1.590	17.750					
50 ALA CB1	1.482	18.380	==				
52 ALA CB1	1.610	17.830					
54 LEU CD1	1.046	26.170	==	==			
54 LEU CD2	1.151	23.180	(==)	==			
57 ILE CD1	0.742	13.640	(!=)		!!		
61 ALA CB1	1.593	18.520	(==)		!!		!!
71 ALA CB1	1.530	18.220	(!!)		==		==
72 ILE CD1	0.697	12.740	==		==		==
77 ILE CD1	0.888	13.390	(==)				
79 LEU CD1	0.903	23.460	==				!!
79 LEU CD2	0.916	25.500	==				!!
80 LEU CD1	1.046	26.580	==				!!
80 LEU CD2	1.011	24.410	==				!!
85 LEU CD1	1.031	24.750	==		==		==
85 LEU CD2	0.927	24.730	==		==		
89 ILE CD1	0.619	14.270	==	==	==		==
90 ILE CD1	1.107	13.940	==	==	==		==
91 ALA CB1	1.544	18.060	==	==			==
92 LEU CD1	1.011	26.780	==	==			==
Methyl	^{1}H	^{13}C	FY	MG	MP	FL	MA
92 LEU CD2	0.915	22.750	==	==			==
93 ILE CD1	0.786	14.460	==	==	==		==

Methyl	^{1}H	^{13}C	FY	MG	MP	FL	MA
100 ALA CB1	1.771	19.750	==		==		==
102 ALA CB1	1.496	18.270	==		==		==
103 ALA CB1	1.470	20.840	==				==
104 ALA CB1	1.442	17.780	==		==		==
107 VAL CG1	0.961	21.610	==	==	==		==
107 VAL CG2	1.031	23.870	==	==	==		==
108 ILE CD1	0.787	10.820	==	==	==		==
112 ALA CB1	1.429	18.440	==		==		!!
114 ALA CB1	1.540	18.000	==				
115 LEU CD1	0.866	25.590	==	==	==		==
115 LEU CD2	0.976	23.090	==	==			==
118 LEU CD1	1.055	26.280					
118 LEU CD2	0.945	22.450	(=!)				
123 LEU CD1	1.062	26.150	==				
123 LEU CD2	0.913	22.100					
127 ALA CB1	1.688	19.170	==		==		==
128 ALA CB1	1.619	17.790					!!
130 VAL CG1	0.976	22.207	==	==	==		==
130 VAL CG2	1.038	23.390	==	==	==		==
133 ILE CD1	0.784	14.630	==	==	==		==
137 LEU CD1	1.036	22.080	==		==		!!
137 LEU CD2	1.088	25.720	==				==
138 LEU CD1	1.089	25.620	==	==	==		!!
138 LEU CD2	1.095	24.580	==	==	==		==
141 ILE CD1	1.022	14.690	==	==	==		==
142 LEU CD1	0.949	26.290	==	==	==		!!
142 LEU CD2	1.063	22.890	==	==			==
144 LEU CD1	0.931	21.430					
144 LEU CD2	0.992	26.440					
146 ILE CD1	0.859	13.100					
147 ILE CD1	0.884	12.910	(==)	==	==		==
149 LEU CD1	0.942	25.480					==
149 LEU CD2	0.850	23.210	==		!!		!!
151 ALA CB1	1.427	19.030	(==)	==	==		==
152 ILE CD1	0.883	13.680	==	==	==		==
156 VAL CG1	0.832	22.100	==		!!		!!
156 VAL CG2	0.826	17.160	==		!!		
157 ILE CD1	0.457	14.070	==	==	==		==
158 LEU CD1	0.976	25.840	==		==		==
159 VAL CG1	0.710	23.450	==		==		==
159 VAL CG2	0.761	21.190	==		==		==
160 ALA CB1	1.387	24.410	==	==	==		==
161 ALA CB1	1.617	18.120	==		==		==
163 LEU CD1	0.670	25.570	(==)				
163 LEU CD2	0.782	24.230	(==)				
169 ALA CB1	1.459	19.220	(==)		!!		
176 VAL CG1	0.600	22.150	==		!!		==
176 VAL CG2	0.914	22.060	==		!!		!!
177 LEU CD1	0.889	26.030	==	==	==		==
177 LEU CD2	0.860	21.700	==	==			

180	ILE CD1	0.740	16.240	==	==	==		==
183	ALA CB1	1.604	20.740	(!!)		==		
192	ILE CD1	0.823	12.790					
194	ALA CB1	1.470	18.450	(==)				
197	LEU CD1	1.011	26.601	==				
199	LEU CD1	0.840	26.133	(!=)				
201	ALA CB1	1.031	22.220	(!!)				
202	ILE CD1	0.731	13.550	==	==	==		==
203	VAL CG1	0.729	19.700	(==)				==
203	VAL CG2	0.499	17.270			==		
208	VAL CG1	0.922	22.860	==		==		==
208	VAL CG2	0.830	19.660	==		==		==
212	VAL CG1	0.925	23.000	==		==		==
212	VAL CG2	0.836	22.820	==		==		==
218	LEU CD1	0.609	26.130	==	==	==		==
218	LEU CD2	0.806	24.900	==	==	==		==
219	ILE CD1	0.580	14.980	==		==		
220	LEU CD1	0.781	24.990	==	==	==		==
220	LEU CD2	0.731	26.220	==	==	==		==
222	ALA CB1	1.568	18.140	==	==	==		==
223	VAL CG1	0.918	20.380	(==)	==			
223	VAL CG2	0.936	21.200	(==)	==	==		==
227	VAL CG1	0.718	21.070	==		==		==
227	VAL CG2	0.818	21.940	==		==		==
229	VAL CG1	0.996	21.470	==	==	==		==
229	VAL CG2	0.786	21.080	==	==	==		==
235	VAL CG1	0.527	21.160	==		==		
235	VAL CG2	1.048	23.110			!!		
236	ILE CD1	0.754	13.530	==		==		
241	ALA CB1	1.594	17.870	(==)				
242	VAL CG1	0.981	21.020	(!=)		!!		
242	VAL CG2	1.167	22.040	(!!)				
246	VAL CG1	1.041	21.100	(==)				
246	VAL CG2	1.075	20.840	(==)				
247	ALA CB1	1.534	19.240	(==)				

ATCase

	Methyl	^{1}H	^{13}C	FY	MG	MP	FL	MA
7	LEU CD1	0.825	24.700	==				
7	LEU CD2	0.779	23.351	==				
9	VAL CG1	0.870	20.219	==	==			
9	VAL CG2	0.879	21.153	==	==			!!
12	ILE CD1	0.486	13.256	(==)		!!	==	==
17	VAL CG1	0.894	20.691	(==)	==			!!
18	ILE CD1	0.734	13.418	==		==		==
21	ILE CD1	0.678	14.203	(==)		==		==
25	ILE CD1	0.848	9.436			!!		
30	LEU CD1	0.851	25.344	==	==			==
30	LEU CD2	0.650	23.065	==	==			==
32	LEU CD1	0.364	23.190	==			==	==
32	LEU CD2	0.286	24.650	==			==	==
35	LEU CD1	0.727	24.537	==	==			==
35	LEU CD2	0.733	21.752	==	==			==
42	ILE CD1	0.676	14.481	!!	==	==		==
44	ILE CD1	0.769	16.259	!!	==	==		==
46	LEU CD2	0.823	22.309	==	==		!!	==
48	LEU CD1	0.844	22.510	(!!)				
48	LEU CD2	0.917	27.064	(!!)				
58	LEU CD1	0.781	24.286	(==)		==		
58	LEU CD2	0.732	26.475	(==)				
59	ILE CD1	0.754	14.045	!=		==		==
61	ILE CD1	0.679	14.380	!=		==		==
66	LEU CD1	0.617	24.699	(==)		!!		!!
66	LEU CD2	0.481	21.671	(==)				
71	VAL CG1	0.790	21.088	==				==
71	VAL CG2	0.902	23.701	==				==
74	LEU CD1	0.789	25.859	(==)		!!	!!	!!
76	LEU CD1	0.788	25.036	==		==		==
76	LEU CD2	0.659	24.234	==		==		==
83	VAL CG1	0.783	21.482	==				==
83	VAL CG2	0.779	21.844	==				==
86	ILE CD1	0.602	11.600	(==)			==	==
91	VAL CG1	0.944	21.777	==	==	==		!!
91	VAL CG2	0.954	21.184	==	==	==		!!
92	VAL CG1	1.032	21.705	==				
92	VAL CG2	0.761	18.600	==		==		
99	LEU CD1	0.739	25.443	(==)	==	==		==
99	LEU CD2	0.751	24.463	(==)	==	==		==
103	ILE CD1	0.794	13.711	==	==	==	==	==
106	VAL CG1	0.905	21.200					==
106	VAL CG2	0.939	19.857	==		==		==
107	LEU CD1	0.448	25.746	==				==
107	LEU CD2	0.533	21.905	==				==
108	VAL CG1	0.764	20.454					
108	VAL CG2	0.822	21.088					
115	ILE CD1	0.858	13.861			!!		
127	VAL CG1	0.696	21.790	==	==	==	!!	==
127	VAL CG2	0.620	20.699	==	==	==	==	==
134	ILE CD1	0.492	13.073	==	==	==	==	==
136	LEU CD1	0.682	25.551	(==)		==		==
136	LEU CD2	0.588	25.695	(==)		==		==
149	VAL CG1	1.161	21.889	==				==
149	VAL CG2	0.954	20.361	==		==		==
150	VAL CG1	0.591	22.056	==		==	!!	==
150	VAL CG2	0.733	22.442	==		==	==	==
151	LEU CD1	0.512	23.053	==				==
151	LEU CD2	0.458	24.279	==				==

MBP

	Methyl	^{1}H	^{13}C	FY	MG	MP	FL
2	ILE CD1	0.402	13.054	(!=)	==	!!	
7	LEU CD1	0.267	25.203	==	==	!!	==
7	LEU CD2	0.615	24.064	==	==		==
8	VAL CG1	0.876	20.962	(!!)	==		
8	VAL CG2	0.923	21.211	(!=)	==		
9	ILE CD1	0.346	14.523	==	==	!!	==
11	ILE CD1	0.107	13.105	==	==	!!	
20	LEU CD1	0.876	24.725	==			
20	LEU CD2	0.638	26.879	==		!!	
23	VAL CG1	0.823	22.314	==	==		
23	VAL CG2	1.252	23.403	==	==		
33	ILE CD1	0.574	10.138	==	==		==
35	VAL CG1	0.331	20.685	==	==		
35	VAL CG2	0.76	22.36	==	==		
37	VAL CG1	0.951	22.84	==	==		
37	VAL CG2	0.766	20.673	==	==		
43	LEU CD1	1.101	26.677	==	==		
43	LEU CD2	1.133	26.431	==	==	!!	

50	VAL	CG1	0.967	21.26	(!!)	==		
50	VAL	CG2	0.906	19.894	(!!)	==		
59	ILE	CD1	0.735	13.79	==	==		
60	ILE	CD1	0.452	14.568	==	==	==	
75	LEU	CD1	0.638	25.725	(!=)	==		
75	LEU	CD2	0.538	20.664	(!!)	==		
76	LEU	CD1	0.897	27.43	==	==		
76	LEU	CD2	0.538	21.891	==	==	==	
79	ILE	CD1	0.161	12.313	==	==	==	
89	LEU	CD1	0.526	26.152	==	==		
89	LEU	CD2	0.484	25.298	==	==		
97	VAL	CG1	0.578	22.085	==	==		==
97	VAL	CG2	1.347	19.363	==	==		==
103	LEU	CD1	0.49	24.748	==	==		
103	LEU	CD2	0.972	24.1	==	==		
104	ILE	CD1	0.845	14.779	==	==		
108	ILE	CD1	0.637	9.981	==	==	==	
110	VAL	CG1	0.767	20.954	==	==		
110	VAL	CG2	0.476	20.424	==	==		
113	LEU	CD1	0.691	26.792				
113	LEU	CD2	0.854	23.625				
115	LEU	CD1	0.684	22.828	(==)			
115	LEU	CD2	0.587	24.597	(!!)			
116	ILE	CD1	0.459	13.706	(!!)			
121	LEU	CD1	0.665	24.729	==			
121	LEU	CD2	0.779	22.791	==			
122	LEU	CD1	0.93	25.548	(!!)			
122	LEU	CD2	0.937	26.848	(!!)			
132	ILE	CD1	0.56	12.36	==			
135	LEU	CD1	0.888	24.04	(!!)			
135	LEU	CD2	0.882	24.317	(=!)			
139	LEU	CD1	0.787	25.914	!=			
139	LEU	CD2	0.852	22.927	!!			
147	LEU	CD1	0.683	22.5	(==)			
147	LEU	CD2	0.936	27.555	(==)			
151	LEU	CD1	0.744	26.232	==			
151	LEU	CD2	0.135	23.579	==		!!	
160	LEU	CD1	-1.003	19.602	(==)	==	==	
160	LEU	CD2	-0.302	25.084	(==)	==	!!	
161	ILE	CD1	0.76	13.081	==	==		==
178	ILE	CD1	0.853	13.905	(==)		==	==
181	VAL	CG1	0.823	21.539	==		==	==
181	VAL	CG2	0.711	22.538	==			
183	VAL	CG1	0.882	22.416	==			==
183	VAL	CG2	0.841	22.42	==		==	==
192	LEU	CD1	1.085	24.249	==	==	==	==
192	LEU	CD2	0.845	25.33	==	==	!!	==
195	LEU	CD1	0.893	23.706	==	==		
195	LEU	CD2	0.845	25.191	==	==		
196	VAL	CG1	0.969	21.649	==	==		
196	VAL	CG2	0.856	23.319	==	==		
198	LEU	CD1	1.018	25.598	(!!)			
198	LEU	CD2	0.813	23.461	(!!)			
199	ILE	CD1	0.743	14.222	==	==		==
212	ILE	CD1	0.867	13.11	(==)			==
226	ILE	CD1	0.06	11.775	==			==
235	ILE	CD1	0.31	13.8			==	
240	VAL	CG1	0.585	20.89	(!=)			
240	VAL	CG2	0.788	21.83	(!!)			==
244	VAL	CG1	1.072	22.083	(!!)			
244	VAL	CG2	0.636	21.606	(!=)			
246	VAL	CG1	0.969	20.441	==			
246	VAL	CG2	0.983	21.596	==		!!	
247	LEU	CD1	0.663	25.833	(=!)			==
247	LEU	CD2	0.645	23.824	(!!)			
259	VAL	CG1	0.925	21.509	==	==		==
259	VAL	CG2	0.98	22.697	==	==		==
261	VAL	CG1	0.679	20.308	==	==		==
261	VAL	CG2	0.565	20.936	==	==		
262	LEU	CD1	0.806	22.681	==	==		
262	LEU	CD2	0.885	25.314	==	==		
266	ILE	CD1	0.915	13.882	==	==		
275	LEU	CD1	0.933	25.202	==	==		==
275	LEU	CD2	0.947	22.493	==	==		==
280	LEU	CD1	0.672	25.779	==	==		==
280	LEU	CD2	0.71	23.405	==	==		
284	LEU	CD1	0.843	23.945	==			
284	LEU	CD2	1.039	27.398	==			
285	LEU	CD1	1.052	25.695	==	==		
285	LEU	CD2	1.119	24.003	==	==		
290	LEU	CD1	1.074	27.886	==	==		
290	LEU	CD2	0.792	21.864	==	==		
293	VAL	CG1	1.004	21.422	==	==		
293	VAL	CG2	0.901	22.246	==	==		
299	LEU	CD1	0.893	26.581				
299	LEU	CD2	0.813	22.958				
302	VAL	CG1	1.059	22.153	==	==		
302	VAL	CG2	1.262	19.07	==	==		
304	LEU	CD1	0.848	24.263	(==)	==		
304	LEU	CD2	0.702	26.586	(==)	==		
311	LEU	CD1	0.87	26.326	==			==
311	LEU	CD2	0.797	22.652	==			==
317	ILE	CD1	1.118	12.483	(==)	==		==
329	ILE	CD1	0.821	12.362	==	==	==	==
333	ILE	CD1	-0.138	12.253	(==)		==	==
343	VAL	CG1	0.863	21.297	==	==	==	==
343	VAL	CG2	0.168	23.236	==	==	==	==
347	VAL	CG1	0.918	20.468	==	==		
347	VAL	CG2	1.043	22.282	==	==		
348	ILE	CD1	0.869	12.965	(==)	==		==
357	VAL	CG1	0.991	21.315	==	==		
357	VAL	CG2	1.045	23.401	==	==	!!	
361	LEU	CD1	0.892	25.264	==	==		==
361	LEU	CD2	0.722	19.605	==	==	==	==
368	ILE	CD1	0.693	15.285	==	==	==	==

MSG

Methyl			^{1}H	^{13}C	FY	MG	MP	FL
5	ILE	CD1	0.820	12.833	(==)			
10	LEU	CD1	0.915	24.486	==	==		
10	LEU	CD2	0.868	22.364	==	==		
12	ILE	CD1	0.008	15.537	==			
20	VAL	CG1	0.463	21.566	==			
20	VAL	CG2	0.739	23.955	==			
24	VAL	CG1	0.541	20.604	==			
24	VAL	CG2	0.137	22.556	==			
25	LEU	CD1	0.295	24.621	==	==		
25	LEU	CD2	-0.285	20.071	==	==		
30	LEU	CD1	0.755	25.838	==			
30	LEU	CD2	0.817	22.144	==			
42	ILE	CD1	0.680	14.704	==	==		
43	VAL	CG1	0.678	20.943	==	==		
43	VAL	CG2	1.051	24.763	==	==		
46	LEU	CD1	0.806	26.301	(==)			

```
 46 LEU CD2  0.827 22.903 (==)
 53 LEU CD1  0.305 25.988 (==)
 53 LEU CD2  0.732 22.305
 54 LEU CD1  1.101 24.942  ==
 54 LEU CD2  0.673 19.976  ==
 60 ILE CD1  0.769 12.120  ==   ==
 64 LEU CD1  0.904 25.428  ==   ==
 64 LEU CD2  0.751 24.554  ==   ==
 75 VAL CG1  0.811 21.936       ==
 75 VAL CG2  0.815 20.637 (==)  ==
 85 LEU CD1 -0.085 24.801  ==   ==
 85 LEU CD2 -0.210 20.081  ==   ==
 88 LEU CD1  0.823 23.547  ==   ==
 88 LEU CD2  0.674 25.333  ==   ==
 91 LEU CD1  0.901 25.353 (!!)
 91 LEU CD2  0.903 24.612 (!!)
 92 VAL CG1  0.759 20.674
 92 VAL CG2  0.683 18.640
 98 VAL CG1  0.852 20.318 (==)
 98 VAL CG2  0.988 22.292
100 VAL CG1  0.958 21.311  ==
100 VAL CG2  0.857 22.218  ==
105 ILE CD1  0.810 10.255 (=!)  !!
109 ILE CD1  0.724 14.490 (==)  !!
117 LEU CD1  0.578 25.348  ==
117 LEU CD2  0.593 26.534  ==
118 VAL CG1  0.230 19.382 (!!)
118 VAL CG2 -0.834 17.697 (!!)     !!
119 VAL CG1  0.737 21.619  ==
119 VAL CG2  0.687 18.891  ==
128 LEU CD1  0.572 24.941 (==)
128 LEU CD2  0.607 24.322 (==)
138 LEU CD1  0.768 23.192 (==)
138 LEU CD2  0.748 26.304 (==)
142 LEU CD1  0.456 27.936  ==   ==
142 LEU CD2 -0.448 21.862  ==   ==
147 ILE CD1  0.262 13.610  ==   ==
148 ILE CD1  0.390 11.341  ==   ==
155 VAL CG1  0.949 21.001
155 VAL CG2  0.934 19.757
166 VAL CG1  0.848 21.207  ==   ==
166 VAL CG2  0.924 22.805  ==   ==
167 ILE CD1  0.831 14.215 (!!)
170 VAL CG1  1.178 22.279  ==   == ==
170 VAL CG2  1.376 25.567  ==   ==
174 LEU CD1  0.842 23.827
178 LEU CD1  0.611 26.784  ==      ==
178 LEU CD2  0.726 26.758  ==      ==
180 LEU CD1 -0.082 24.390  ==
180 LEU CD2  0.359 22.737  ==
188 VAL CG1  0.407 21.750  ==   ==
188 VAL CG2  0.743 23.700  ==   ==
189 VAL CG1  0.734 21.345       ==
189 VAL CG2  0.541 18.759  ==   ==
193 VAL CG1  0.703 19.420  ==   ==
193 VAL CG2  0.205 19.366  ==   ==
194 VAL CG1  0.860 20.417       ==
194 VAL CG2  0.773 20.321 (!=)  ==
198 LEU CD1  0.663 22.639  ==   ==
198 LEU CD2  0.813 26.102  ==   ==
200 ILE CD1  0.746 16.119  ==   == ==
202 LEU CD1  0.768 24.801  ==
202 LEU CD2  0.501 22.680  ==
210 LEU CD1  1.090 28.067  ==      ==
210 LEU CD2  0.904 22.536  ==      ==
217 VAL CG1  0.800 21.989  ==
217 VAL CG2  0.880 21.929  ==
229 ILE CD1  0.562 10.616  ==   == ==
230 LEU CD1  0.680 21.754  ==
230 LEU CD2 -0.499 24.072 (==)
231 LEU CD1  0.876 25.413  ==
231 LEU CD2  1.069 24.834  ==
236 LEU CD1  0.804 25.156 (==)  ==
236 LEU CD2  0.699 22.500 (==)  ==
238 ILE CD1  0.503 13.747  ==   == ==
240 LEU CD1  0.632 24.913  ==   == ==
240 LEU CD2  0.715 24.407  ==   ==
242 ILE CD1  0.664 12.365  ==      ==
248 ILE CD1  0.756 12.276 (!!)
256 ILE CD1  0.901 11.691  ==      ==
259 VAL CG1  0.651 19.992  ==   ==
259 VAL CG2  0.648 21.191  ==   == ==
260 ILE CD1  0.651 11.138 (==)  == !!
261 VAL CG1  0.898 22.279  ==   ==
261 VAL CG2  0.924 21.020  ==   ==
265 ILE CD1   0.378 9.490 (==)  ==
268 ILE CD1  0.655 13.602 (==)     !!
269 LEU CD1  0.876 26.885  ==
269 LEU CD2  0.655 22.419  ==
275 VAL CG1  0.833 23.138 (==)
275 VAL CG2  0.785 21.430 (==)
278 VAL CG1  0.423 21.032 (==)
278 VAL CG2  0.642 18.142 (==)
284 ILE CD1  0.745 13.198 (==)
285 LEU CD1  0.945 24.614
285 LEU CD2  0.895 23.633
286 LEU CD1  0.881 25.696 (==)
286 LEU CD2  0.768 26.157 (==)
291 LEU CD1  0.601 25.289  ==   ==
291 LEU CD2  0.771 26.260  ==   ==
293 LEU CD1  0.930 25.478 (!=)
293 LEU CD2  0.778 24.044 (=!)
298 LEU CD1  1.052 23.575 (!=)
298 LEU CD2  0.821 25.239 (==)
309 ILE CD1  0.791 13.228
310 VAL CG1  0.753 21.150
310 VAL CG2  0.854 20.859
313 LEU CD1  0.938 25.130 (!=)
313 LEU CD2  0.807 24.015 (==)
327 ILE CD1  0.572 13.073  ==
329 LEU CD1  0.204 25.272 (==)
329 LEU CD2  0.625 22.249  ==
334 LEU CD1  0.835 21.446       ==
334 LEU CD2  0.780 27.669 (==)  ==
335 LEU CD1  0.818 25.213 (==)
335 LEU CD2  0.633 22.086 (!!)
337 ILE CD1  0.875 14.606  ==   ==
340 VAL CG1  1.022 20.603          !!
340 VAL CG2  0.865 18.883
343 LEU CD1  0.988 22.988  ==
343 LEU CD2  0.745 25.785  ==
346 ILE CD1  0.598 13.843
348 VAL CG1  0.163 20.967  ==   ==
348 VAL CG2  0.402 21.448  ==   ==
349 ILE CD1  0.778 14.919  ==   ==
361 ILE CD1  0.999 12.815  ==   == !!
362 LEU CD1  1.105 24.066  ==   ==
362 LEU CD2  0.769 27.381  ==   ==
```

365	VAL	CG1	-0.085	20.364	==	==	
365	VAL	CG2	0.566	21.867	==	==	
370	ILE	CD1	0.871	13.820	==	==	
372	LEU	CD1	0.863	23.064	==		
372	LEU	CD2	1.033	25.825	==		!!
375	LEU	CD1	0.885	25.628	==	==	
375	LEU	CD2	0.721	22.069	==	==	
377	VAL	CG1	1.076	21.349			
377	VAL	CG2	1.215	20.579			
386	VAL	CG1	0.670	19.406	==	==	
386	VAL	CG2	0.726	21.071	==	==	
388	ILE	CD1	0.290	12.747	==	==	
389	VAL	CG1	0.720	22.383	(==)		
389	VAL	CG2	0.437	20.605	(==)		
399	VAL	CG1	0.912	21.973	==		
399	VAL	CG2	0.989	20.715	==		
409	ILE	CD1	0.548	13.073	==	==	
413	LEU	CD1	0.718	26.081	==	==	
413	LEU	CD2	0.807	22.095	==	==	
420	LEU	CD1	0.469	24.802	==	==	
420	LEU	CD2	0.373	22.504	==	==	
424	ILE	CD1	0.745	14.922	==	==	==
433	LEU	CD1	0.971	27.191	==		
433	LEU	CD2	0.888	25.452	==		
435	LEU	CD1	1.194	27.524	==	==	
435	LEU	CD2	0.801	21.476	==	==	==
439	ILE	CD1	0.790	13.782	==		!!
446	VAL	CG1	0.980	21.292	==	==	
446	VAL	CG2	1.134	22.836	==	==	
449	ILE	CD1	0.642	14.145	==		!!
454	LEU	CD1	0.387	24.883			
454	LEU	CD2	0.452	21.609			
464	VAL	CG1	0.474	20.503	==	==	
464	VAL	CG2	0.670	17.943	==	==	
471	LEU	CD1	0.916	25.988		==	
471	LEU	CD2	1.075	22.194	(==)	==	
482	ILE	CD1	0.648	14.308			
490	VAL	CG1	0.904	19.622	==	==	
490	VAL	CG2	0.854	20.949	==	==	
491	LEU	CD1	0.561	25.082	==	==	
491	LEU	CD2	0.282	20.686	==	==	
494	LEU	CD1	0.627	25.945	==	==	
494	LEU	CD2	0.670	22.337	==	==	
498	LEU	CD1	0.761	25.082	==	==	==
498	LEU	CD2	0.619	23.324	==	==	==
504	ILE	CD1	0.722	13.295	==	==	
514	LEU	CD1	1.066	25.980	==		
514	LEU	CD2	0.937	22.371	==		
526	LEU	CD1	0.115	23.498	(==)		
526	LEU	CD2	0.100	22.406	(==)		==
535	VAL	CG1	1.134	24.923			
535	VAL	CG2	0.916	17.974	==		
543	LEU	CD1	0.572	25.180	==		
543	LEU	CD2	0.250	21.491	==		
546	LEU	CD1	0.781	25.237	(==)		
546	LEU	CD2	0.261	20.492	(==)		
553	VAL	CG1	1.087	22.981	(==)		
553	VAL	CG2	1.115	20.128	(==)		
556	VAL	CG1	0.894	21.888	(==)		
556	VAL	CG2	0.865	20.935	(=!)		
560	ILE	CD1	0.603	13.815	==	==	
572	LEU	CD1	0.721	26.490	==	==	
572	LEU	CD2	1.055	23.384	==	==	
573	LEU	CD1	0.989	24.348	==		
573	LEU	CD2	0.950	25.639	==		
576	LEU	CD1	0.826	24.877	==	==	
576	LEU	CD2	1.214	24.663	==	==	
577	LEU	CD1	0.781	26.739	(!!)		
577	LEU	CD2	0.738	22.528	(!!)		
579	ILE	CD1	0.757	14.656	==	==	==
581	VAL	CG1	0.530	20.609	==	==	
581	VAL	CG2	0.527	20.334	==	==	
592	ILE	CD1	0.825	13.646	==		==
596	LEU	CD1	1.050	26.922	==		==
596	LEU	CD2	1.053	24.904	==		
600	VAL	CG1	0.752	22.462	==		==
600	VAL	CG2	0.974	25.068	==		
603	ILE	CD1	0.502	14.352	(==)		==
604	LEU	CD1	0.912	27.761	==		
604	LEU	CD2	0.754	23.071	==		
607	VAL	CG1	1.033	23.104	==		==
607	VAL	CG2	1.120	26.709	==		==
608	VAL	CG1	1.329	20.748	(==)		
608	VAL	CG2	1.155	21.975	(==)		
611	VAL	CG1	0.864	21.774	==		
611	VAL	CG2	1.349	25.146	==		==
615	ILE	CD1	0.772	12.580			
620	VAL	CG1	0.945	22.917			
623	ILE	CD1	0.784	14.613	(==)		
626	VAL	CG1	0.805	20.784	==		
626	VAL	CG2	0.882	21.600	==		
628	LEU	CD1	0.894	25.544	==		
628	LEU	CD2	0.678	23.460	==		
635	LEU	CD1	0.867	27.517	==		
635	LEU	CD2	1.020	25.306	==		
642	ILE	CD1	0.647	15.941	==		==
646	LEU	CD1	0.863	23.659	==		
646	LEU	CD2	0.829	25.235	==		
650	ILE	CD1	0.581	8.057	==		==
651	LEU	CD1	1.193	28.466	==		==
651	LEU	CD2	1.057	25.318	==		==
656	VAL	CG1	0.763	22.427	==		==
656	VAL	CG2	1.119	24.262	==		==
660	LEU	CD1	0.717	25.900	==		
660	LEU	CD2	0.479	23.362	==		
666	VAL	CG1	0.899	19.705	==		
666	VAL	CG2	1.127	21.958	(==)		
667	VAL	CG1	1.058	22.628	==		
667	VAL	CG2	1.131	23.404	==		
696	LEU	CD1	0.738	26.693	==		
696	LEU	CD2	0.903	24.144	==		
697	ILE	CD1	0.574	14.757	(==)		==
699	LEU	CD1	0.907	25.034	==		
699	LEU	CD2	0.881	22.156	==		
701	VAL	CG1	1.046	21.452			==
701	VAL	CG2	1.035	19.731			
711	LEU	CD1	0.806	25.811	==		
711	LEU	CD2	1.022	22.836	==		
712	LEU	CD1	0.182	24.727	==		
712	LEU	CD2	0.057	22.944	==		!!
717	LEU	CD1	0.980	25.176	(==)		
717	LEU	CD2	0.956	23.695	(==)		
724	LEU	CD1	0.938	24.788			
724	LEU	CD2	0.875	22.968			

a7a7

Methyl	^{1}H	^{13}C	FY	MG	MP	FL
14 VAL CG1	0.867	17.770				
21 LEU CD1	0.607	25.996	(!=)	==	==	==
24 VAL CG1	0.930	24.215	==	==		==
24 VAL CG2	0.873	21.800	==	==	==	==
31 VAL CG1	0.975	21.101	==	==	==	==
38 LEU CD1	0.639	26.082	==	==		==
38 LEU CD2	0.701	28.507	==	==	==	==
46 VAL CG1	0.821	19.945	==	==	==	==
46 VAL CG2	0.938	22.010	==	==	==	==
47 LEU CD1	0.714	25.259	==	==	==	==
47 LEU CD2	0.572	28.341	==	==	==	!!
48 LEU CD1	0.608	26.717	==	==	==	==
48 LEU CD2	0.748	23.970	==	==	==	!!
49 ILE CD1	0.594	14.168	==	==	==	==
54 VAL CG1	0.923	20.875	==	==	==	==
54 VAL CG2	0.918	21.440	==	==	==	==
58 LEU CD1	0.440	21.343	(!!)	==		==
58 LEU CD2	0.784	26.121	(!=)	==	==	==
59 ILE CD1	0.791	13.464	(==)	==	==	==
64 ILE CD1	0.799	12.983	==	==	==	==
67 ILE CD1	0.542	13.905	==	==	==	==
69 LEU CD1	0.755	24.774	==	==	==	==
69 LEU CD2	0.425	22.438	==	==	==	==
70 ILE CD1	0.660	11.698	==	==	==	==
74 VAL CG1	0.770	20.747	==	==	==	!!
74 VAL CG2	0.947	22.071	==	==	==	!!
77 VAL CG1	0.733	22.491	==	==	==	==
77 VAL CG2	0.755	21.784	==	==		==
81 LEU CD1	1.077	22.548	==	==	==	==
81 LEU CD2	1.194	26.063	==	==	==	==
82 VAL CG1	1.173	21.933	==	==	==	==
82 VAL CG2	1.099	21.877	==	==	==	==
87 VAL CG1	1.033	20.901	(==)	==		!!
87 VAL CG2	1.151	20.479	(==)	==	!!	!!
88 LEU CD1	0.242	25.077	==	==	==	!!
88 LEU CD2	-0.095	20.700	==	==	==	!!
89 VAL CG1	0.784	21.464	==	==		!!
89 VAL CG2	0.711	23.461	==	==	==	!!
94 ILE CD1	0.793	13.229	(==)		==	==
101 VAL CG1	0.879	20.980				
101 VAL CG2	0.857	21.657				
106 LEU CD1	0.440	24.657	==	==	==	==
106 LEU CD2	0.227	23.969	==	==	==	==
107 VAL CG1	0.828	22.135	==		==	!!
107 VAL CG2	0.745	21.775	==			!!
109 ILE CD1	0.726	13.179	==	==	==	==
112 LEU CD1	0.652	23.297	==	==	==	==
112 LEU CD2	0.634	26.192	==	==	==	==
113 VAL CG1	0.301	20.996	==	==	==	==
113 VAL CG2	-0.033	21.787	==	==	==	==
116 VAL CG1	0.950	22.011	==	==	==	!!
116 VAL CG2	0.940	21.613	==	==	==	!!
129 VAL CG1	0.283	18.799				
129 VAL CG2	0.139	18.249			==	
134 VAL CG1	0.742	22.670	==	==	==	==
134 VAL CG2	0.872	23.838	==	==	==	==
136 LEU CD1	0.667	27.678	==	==	==	!!
136 LEU CD2	0.410	26.611	==	==	==	!!
137 ILE CD1	0.374	14.071	==	==	==	==
141 ILE CD1	0.616	12.458	==	==	==	==
144 ILE CD1	0.762	11.757	==			==
148 LEU CD1	0.731	25.245	==	==	==	==
148 LEU CD2	0.454	26.003	==	==	==	==
157 ILE CD1	0.638	13.663	(==)	==	==	==
165 ILE CD1	0.425	13.629	==	==	==	==
172 VAL CG1	0.696	23.655	==	==	==	==
172 VAL CG2	0.799	22.860	==	==	==	==
173 VAL CG1	0.946	21.461	==	==	==	==
173 VAL CG2	0.982	23.532	==	==	==	==
176 LEU CD1	0.638	25.759	(==)	==	==	==
176 LEU CD2	0.901	22.386	(==)	==	==	==
184 LEU CD1	0.733	25.965	==	==	==	==
184 LEU CD2	0.806	22.537	==	==	==	==
190 VAL CG1	-0.176	21.380	==	==	==	==
190 VAL CG2	0.630	23.016	==	==	==	==
192 LEU CD1	1.077	24.522	==	==	==	==
192 LEU CD2	0.975	26.319	==	==	==	==
194 ILE CD1	0.506	10.361	==	==	==	==
197 LEU CD1	0.645	23.729	==	==	==	==
197 LEU CD2	0.652	25.212	==	==	==	==
201 LEU CD1	0.784	25.766	==	==	==	==
201 LEU CD2	0.660	22.016	==	==	==	==
207 LEU CD1	0.835	24.814	==	==	==	==
207 LEU CD2	0.762	26.319	==	==	==	==
212 ILE CD1	0.403	14.420	==	==	==	==
215 ILE CD1	1.051	16.367	==	==	==	==
217 VAL CG1	0.916	20.710	==	==		!!
217 VAL CG2	0.945	21.220	==	==	==	!!
223 ILE CD1	0.782	11.794	==	==	==	==
229 VAL CG1	1.023	21.420	==	==	==	==
229 VAL CG2	1.180	23.133	==	==	==	==
233 LEU CD1	0.872	25.610	==	==	==	==
233 LEU CD2	0.696	22.819	==	==	==	==

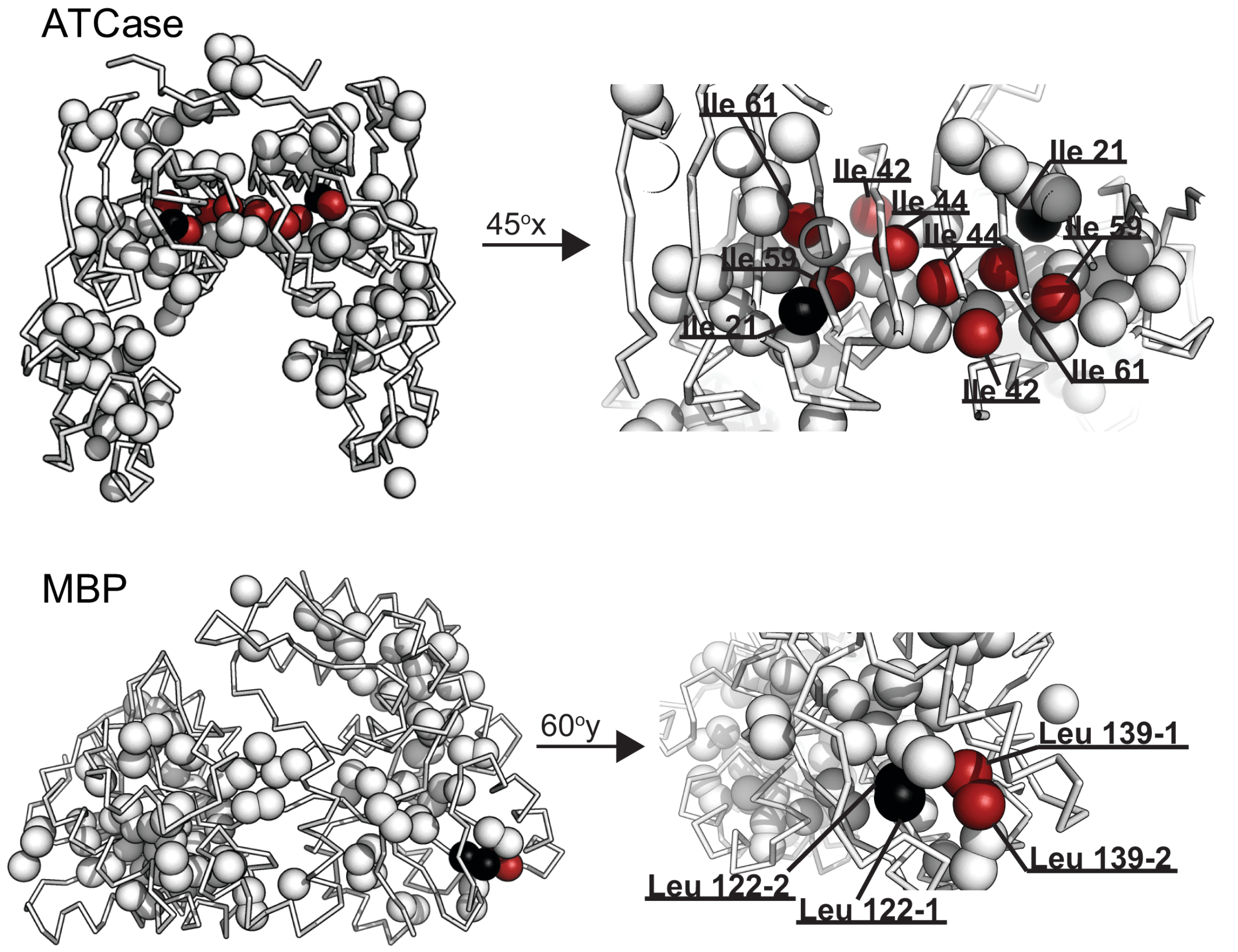

Supplementary Fig. 4 Sources of errors in the automatic methyl resonance assignments generated by MethylFLYA. Carbon atoms of the erroneously assigned methyls are shown as red spheres, whereas their correct assignment positions are given in black spheres, or exceptionally in red when the assignment at those positions is also incorrect (i.e. for assignment swaps such as Ile42↔Ile44). The mis-assigned resonances belong to nearby methyl groups. For ATCase, the assignment errors cluster at the interface of the two subunits of the homodimer. The erroneously assigned methyls are labeled on both sides of the dimer interface.

Supplementary Table 2 Summary of errors in the automatic methyl resonance assignments generated by MethylFLYA. The mis-assigned methyls are assigned to spatially proximal residues.

Protein	Methyl group	Erroneously assigned to	C–C distance (Å) between correct and erroneous assignment
ATCase	Ile 42 δ_1	Ile 44 δ_1	5.5
	Ile 44 δ_1	Ile 42 δ_1	5.5
	Ile 59 δ_1	Ile 21 δ_1	4.0
	Ile 61 δ_1	Ile 59 δ_1	5.9
MBP	Leu 139 δ_1	Leu 122 δ_1	5.2
	Leu 139 δ_2	Leu 122 δ_2	6.9

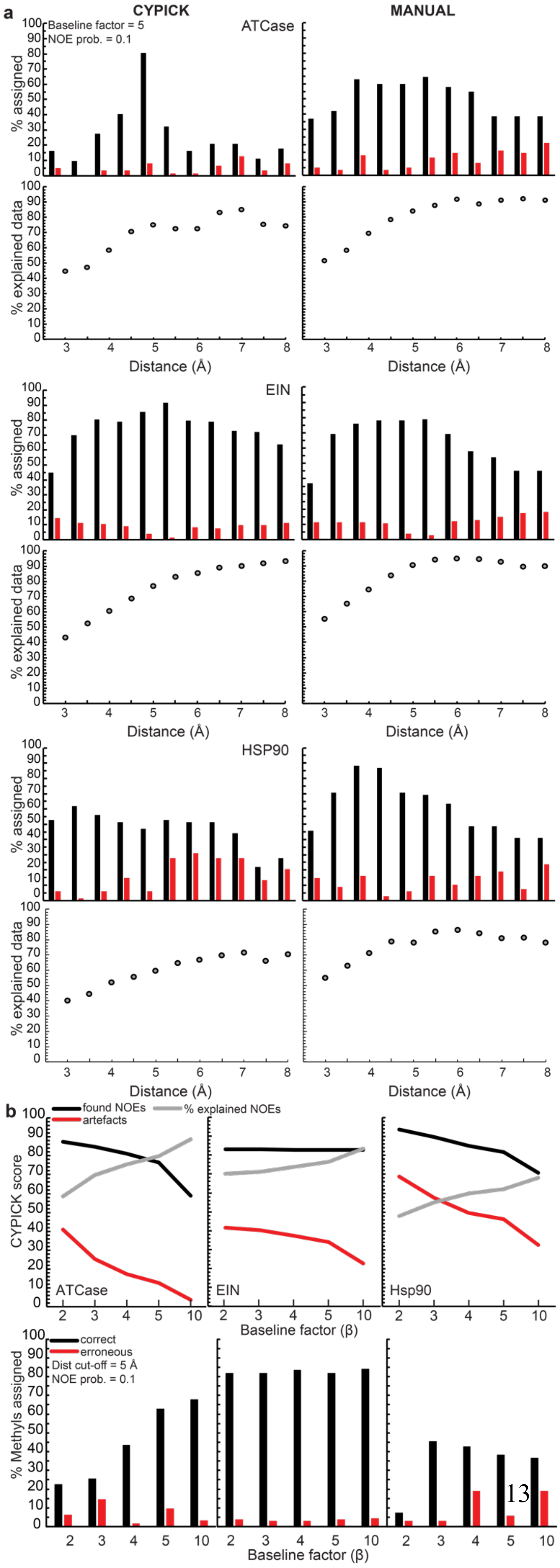

Supplementary Fig. 5 Parameter optimization for automatic NOESY peak picking with CYPICK. **a** Percentage of accurately (black) and erroneously (red) assigned methyls as a function of methyl ^{1}H–^{1}H NOE distance cutoffs, d_{cut}, at fixed NOE probability value p_{NOE} = 0.1. Results using the automatically generated CYPICK NOESY lists are in the left columns, and results obtained using the manually prepared NOESY lists are in the right column. **b** Varying baseline factors for automatic peak picking of methyl-methyl NOESY spectra using CYPICK at fixed distance cutoff d_{cut} = 5 Å and NOE probability value p_{NOE} = 0.1. Variation of the CYPICK find score (*black*), artifact score (red), and the percentage of explained inter-methyl NOEs (*grey*) as a function of the baseline factor. For all three proteins, a fixed distance cutoff d_{cut} = 5 Å and an NOE observation probability p_{NOE} = 0.1 were used to generate the expected methyl-methyl NOEs. Assignment percentages are relative to the number of reference assignments.

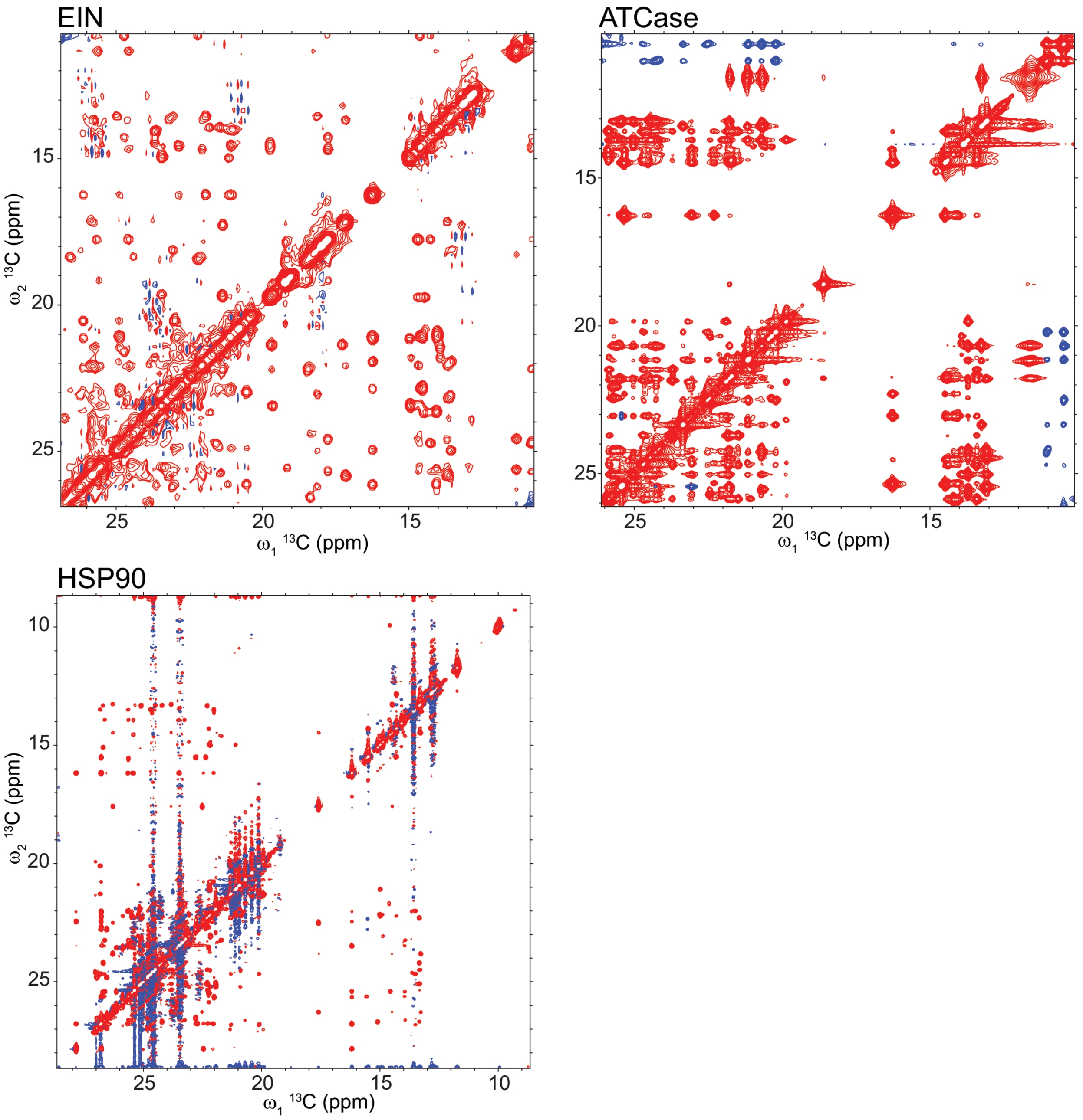

Supplementary Fig. 6 2D $^{13}C(\omega_1)$-$^{13}C(\omega_2)$ projections of 3D CCH NOESY (ATCase, HSP90) or 4D HCCH NOESY spectra (EIN). All spectra are plotted with contour levels starting at a signal-to-noise ratio of three, as determined by the software Sparky[5]. Positive and negative contours are colored in red and blue, respectively. The spectra were acquired previously by Venditti *et al.*[6] (EIN), Velyvis *et al.*[7] (ATCase), and Shah *et al.*[8] (HSP90).

Supplementary Table 3 MethylFLYA computation times (h) for different combinations of input NMR data, as in Fig. 3.

Protein	Filtered NOEs	Unfiltered NOEs	L, V=LV	2L, 2V	L, V=LV, 2LV
EIN	0.52	0.50	0.54	0.48	0.54
ATCase	0.38	0.40	0.40	0.36	0.41
MBP	0.46	0.45	0.53	0.52	0.59
MSG	1.23	1.23	1.53	1.20	1.24
$\alpha_7\alpha_7$	0.50	0.51	0.60	0.69	0.79

Calculations were performed using 100 Intel Xeon E5-2690 processor cores in parallel.

Supplementary Table 4 Results of the CYPICK application to the 3D CCH NOESY (ATCase, HSP90) and 4D HCCH NOESY (EIN) spectra. The results are given for the baseline factor $\beta = 5$. A find score indicates which percentage of NOE peaks from the reference were also found by CYPICK. Overall, find, and artifact scores are defined by Würz *et al.*[9]

Protein	Peaks (reference)	Peaks (CYPICK)	Overall score (%)	Find score (%)	Artifact score (%)
EIN	618	775	74	83	34
ATCase	563	495	74	77	13
HSP90	409	624	68	82	46

Supplementary Table 5 Description of the information provided for the MethylFLYA runs using minimal data input

Data set	Peak picking method	Information provided	Explanation
**m1**	Manual	**i)** Ile, Ala and ambiguous Leu/Val methyl resonance type<br>**ii)** methyl-methyl NOEs picked with no knowledge of the reference ^{1}H-^{13}C positions in the 2D HMQC spectrum	**i)** best guess assignment of methyl residue types based on the number expected from protein sequence and BMRB chemical shift statistics<br>**ii)** automatic assignment of methyl-methyl NOE types using CYANA, based on **i)**
**m2**		**i)** Ile, Ala, ambiguous Leu/Val peak type, and ambiguous Ala/Leu/Val type for a subset of methyl resonances<br>**ii)** methyl-methyl NOEs picked with no knowledge of the reference ^{1}H-^{13}C positions in the 2D HMQC spectrum	**i)** the same as in *m1;* with additional ambiguity in assignment of residue types of the methyl resonances in the area of the overlap between residue types based on the BMRB chemical shift statistics (Supplementary Fig. 7A,B)<br>**ii)** automatic assignment of methyl-methyl NOE types using CYANA, based on **i)**
**m3**		**i)** Ile, Ala and ambiguous Leu/Val methyl resonance type<br>**ii)** methyl-methyl NOEs picked with no knowledge of the reference ^{1}H-^{13}C positions in the 2D HMQC spectrum<br>**iii)** geminal methyl pairing for completely resolved Leu/Val methyl resonances	**i)** the same as in *m1*<br>**ii)** automatic assignment of methyl-methyl NOE types using CYANA, based on **i)**<br>**iii)** geminal pairing of Leu/Val methyl resonances of the same residue restricted to well-resolved peaks in the 2D [^{1}H,^{13}C]-HMQC spectrum (Supplementary Fig. 7C)
**cy1**	Automated (CYPICK)	**i)** Ile, Ala and ambiguous Leu/Val methyl resonance type<br>**ii)** methyl-methyl NOEs picked automatically using CYPICK with no knowledge of the reference ^{1}H-^{13}C positions in the 2D HMQC spectrum; the generated NOESY peak list filtered based on the CYPICK generated ^{1}H-^{13}C peak list for the 2D HMQC spectrum	**i)** best guess assignment of the methyl residue types based on the expected number of methyl resonances from the protein sequence and the BMRB chemical shift statistics<br>**ii)** automatic assignment of methyl-methyl NOE types using CYANA, based on **i)**
**cy2**		**i)** Ile, Ala, ambiguous Leu/Val peak type, and ambiguous Ala/Leu/Val type for a subset of methyl resonances<br>**ii)** methyl-methyl NOEs picked automatically using CYPICK with no knowledge of the reference ^{1}H-^{13}C positions in the 2D HMQC spectrum; the generated NOESY peak list filtered based on the CYPICK generated ^{1}H-^{13}C peak list for the 2D HMQC spectrum	**i)** the same as in *cy1;* with additional ambiguity in assignment of residue types of the methyl resonances in the area of the overlap between residue types based on the BMRB chemical shift statistics (Supplementary Fig. 7B)<br>**ii)** automatic assignment of methyl-methyl NOE types using CYANA, based on **i)**
**cy3**		**i)** Ile, Ala and ambiguous Leu/Val methyl resonance type<br>**ii)** methyl-methyl NOEs picked automatically using CYPICK with no knowledge of reference ^{1}H-^{13}C positions in the 2D HMQC spectrum; the generated NOESY peak list filtered based on the CYPICK generated ^{1}H-^{13}C peak list for the 2D HMQC spectrum<br>**iii)** geminal methyl pairing for completely resolved Leu/Val methyl resonances; using CYPICK generated ^{1}H-^{13}C peak list	**i)** the same as in *cy1*<br>**ii)** automatic assignment of methyl-methyl NOE types using CYANA, based on **i)**<br>**iii)** the geminal pairing of Leu/Val methyl resonances belonging to the same residue restricted only to the well-resolved peaks in the CYPICK-picked 2D [1H,13C]-HMQC spectrum (Supplementary Fig. 7C)

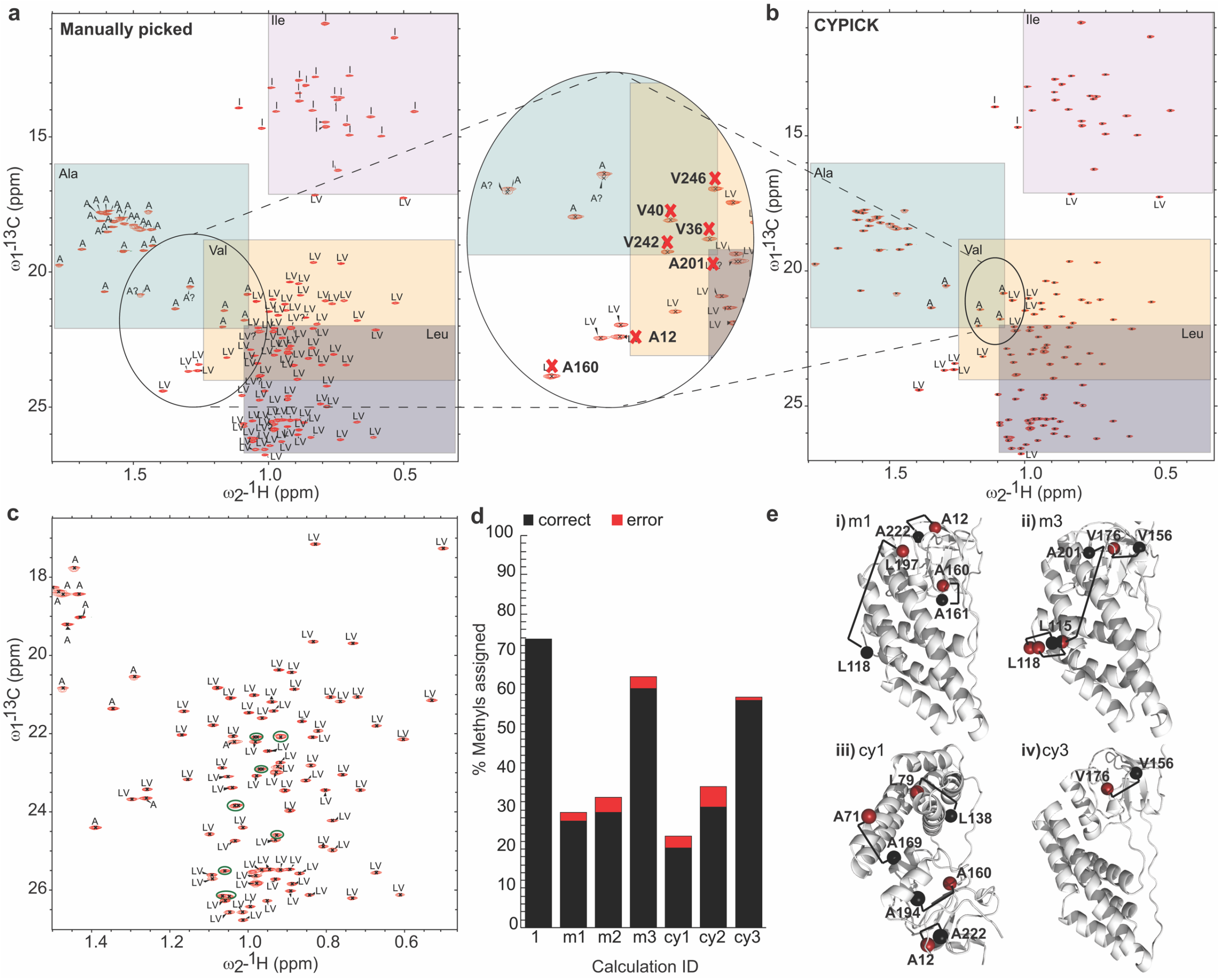

Supplementary Fig. 7 Summary of the results of methyl resonance assignment of EIN using MethylFLYA with no knowledge of the reference ^{1}H-^{13}C resonance positions and methyl residue types, as detailed in Supplementary Table 5. **a** The 2D [^{1}H,^{13}C]-HMQC spectrum of EIN with indicated manually picked resonances. The indicated methyl residue types were attributed based on the full BMRB chemical shift statistics. In the inset on the right, peaks with incorrectly attributed residue types, based on the known reference assignment, are indicated (red 'X'). **b** The 2D [^{1}H,^{13}C]-HMQC spectrum of EIN with the methyl resonances picked automatically using CYPICK indicated. The circled area (linked to the inset) points to the incorrectly attributed residue types based on the strategy described in Supplementary Table 5 and Methods. The resonances in the circled area were either attributed the "best guess" residue type assignment (Supplementary Table 5, data sets m1, cy1) or an ambiguous Ala or Leu/Val type assignment (data sets m2, cy2). **c** Part of the 2D [^{1}H,^{13}C]-HMQC spectrum from (A) with LV resonances that were attributed geminal pairs indicated with both black "X" and the "LV" label. The overlapped resonances that were not attributed their geminal pairs, and the resonances for which the reference geminal assignment was not available, are circled in green. Note the correction in the attributed residue types compared to (A)**,** which is based on this additional (geminal pairing) information. **d** MethylFLYA performance using the input data sets detailed in Supplementary Table 5. The first bar (labeled '1') is included for reference and corresponds to the performance of MethylFLYA

when using maximal input information as given in Fig. 2 and Table 1. **e** Erroneously assigned methyls depicted on the crystal structure of EIN (PDB ID: 1EZA) when using four different data sets given in Supplementary Table 5. **i)** Data set m1. The erroneously assigned methyls shown as red spheres are assigned to (':') the resonances of the methyls shown as black spheres and connected with a black line. These are: Ala160 : Ala161, Ala12 : Ala222, L197$_{\delta1}$: L118$_{\delta1}$. Note that the errors in assignment of Ala12 and Ala160 could have been predicted *a priori*, as the methyl resonances corresponding to Ala12 and Ala160 get incorrectly classified as methyl resonances of Leu/Val type (see Supplementary Fig. 8). If those methyl assignments were not considered, the only remaining error would be L197$_{\delta1}$: L118$_{\delta1}$. **ii)** Data set m3. The indicated errors are: Val176$_{\gamma2}$: Val156$_{\gamma1}$, Leu115$_{\delta1}$: Ala201, Leu118$_{\delta1/\delta2}$: Leu115$_{\delta1/\delta2}$. **iii)** Data set cy1. The indicated errors are: Ala71 : Ala169, Leu79$_{\delta2}$: Leu138$_{\delta1}$, Ala160 : Ala 194, Ala12: Ala 222. Note that the errors in assignment of Ala12 and Ala160 could have been predicted *a priori*, as the methyl resonances corresponding to Ala12 and Ala160 get incorrectly classified as methyl resonances of Leu/Val type (see Supplementary Fig. 8). If those methyl assignments were not considered, the remaining errors would be Ala71 : Ala169 and Leu79$_{\delta2}$: Leu138$_{\delta1}$. **iv)** Data set cy3. The indicated error is: Val176$_{\gamma2}$: Val156$_{\gamma1}$.

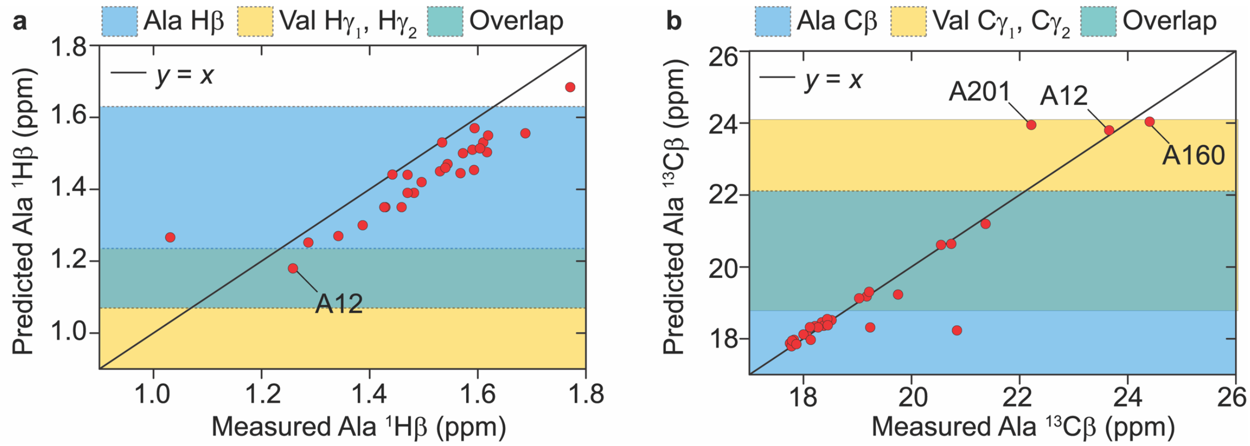

Supplementary Fig. 8 Correlations between the measured alanine methyl chemical shifts for EIN and those predicted with SHIFTX2[10] based on the crystal structure (PDB ID: 1EZA). **a** Methyl proton chemical shifts measured (x-axis) and predicted (y-axis). **b** Methyl carbon chemical shifts measured (x-axis) and predicted (y-axis). The labels indicate the alanine methyl chemical shifts that fall in the area of Ala/Val overlap or the area of Val methyl shifts based on the full BMRB chemical shift statistics.

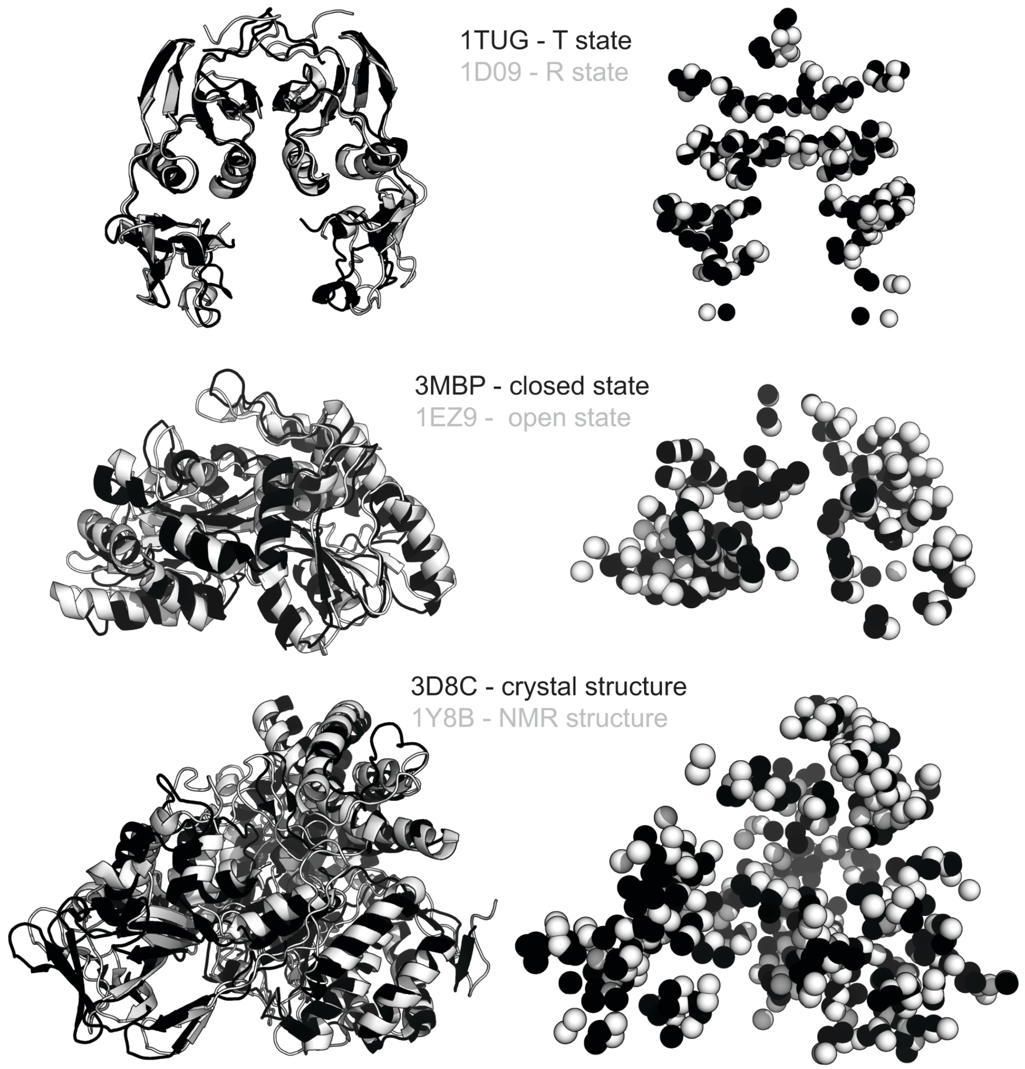

Protein	PDB ID	correct	error
atcase	1d09 (Rstate)	35	4
	1tug (Tstate)	36	1
mbp	1ez9 (open)	83	2
	3mbp (closed)	75	2
msg	1y8b (NMR)	129	0
	1d8c (X-ray)	173	0

Supplementary Fig. 9 MethylFLYA performance on different input structures for three enzymes in the benchmark. Differences in backbone conformations between the different protein states are shown with protein structures in cartoon representation (*right* column). In the *left* column, the positions of the methyl carbons are indicated with spheres for each of the conformers, with colors matching those assigned to the backbone (*right*). The total number of accurate and erroneous "strong" methyl assignments generated with MethylFLYA using different conformers is summarized in the table (bottom row).

Supplementary Table 6 Results of the MAGIC protocol[4] runs on ATCase, EIN, and HSP90, using, where applicable, both filtered and unfiltered NOESY peak lists. The protocol was run for a range of distance thresholds (lower and upper) and peak matching tolerances (^{13}C, ^{1}H). The assignment was considered accurate if the chemical shift was assigned within 0.4 ppm in ^{13}C and 0.04 ppm ^{1}H of the known reference assignment. The best scoring runs, based on the MAGIC score, are highlighted in grey and green for the filtered and unfiltered NOESY lists, respectively.

Protein data set	Labeled methyl groups			Dist. (Å) lower–upper	Tolerance ^{13}C, ^{1}H	Calc. ID	MAGIC confident	Confident Correct	Confident Error	Confident No ref.	Ambig.	MAGIC score
	All	With reference assign-ment	With NOESY peaks									
EIN filtered	146	133	116	4–7	0.1, 0.01	--	*NA**	--	--	--	--	--
				5–8	0.1, 0.01	--	*NA**	--	--	--	--	--
				6–9	0.1, 0.01	1	85	35	48	2	1	152.357
				7–10	0.1, 0.01	2	85	71	13	1	8	204.786
					0.2, 0.02	--	*NA**	--	--	--	--	--
					0.4, 0.04	--	*NA**	--	--	--	--	--
EIN unfiltered	146	133	NA	7–10	0.1, 0.01	3	90	80	9	1	7	224.037
					0.2, 0.02	--	*NA**	--	--	--	--	--
					0.4, 0.04	--	*NA**	--	--	--	--	--
ATCase (R) filtered	66	62	54	4–7	0.1, 0.01	4	34	4	28	2	6	80.171
				5–8	0.1, 0.01	5	42	2	36	4	4	129.474
				6–9	0.1, 0.01	6	38	4	31	3	4	147.295
				7–10	0.1, 0.01	7	47	39	6	2	1	246.549
					0.2, 0.02	8	32	2	27	3	11	187.531
					0.4, 0.04	9	36	5	28	3	3	128.384
ATCase (R) unfiltered	66	62	NA	7–10	0.1, 0.01	10	50	29	19	2	1	275.089
					0.2, 0.02	11	43	6	34	3	3	248.314
					0.4, 0.04	12	38	10	25	3	6	228.797
ATCase (T) filtered	66	62	54	7–10	0.1, 0.01	13	34	5	27	2	2	140.034
					0.2, 0.02	14	41	7	31	3	4	178.45
					0.4, 0.04	15	43	7	33	3	0	138.833
ATCase (T) unfiltered	66	62	NA	7–10	0.1, 0.01	16	50	37	12	1	1	299.539
					0.2, 0.02	17	48	36	10	2	1	321.919
					0.4, 0.04	18	44	8	33	3	0	183.168
Hsp90-N unfiltered	76	72	71	4–7	0.1, 0.01	19	39	6	30	3	4	113.516
				5–8	0.1, 0.01	20	43	22	20	1	3	132.423
				6–9	0.1, 0.01	21	43	12	29	2	5	172.257
				7–10	0.1, 0.01	22	56	20	31	5	4	193.899
					0.2, 0.02	23	55	23	29	3	2	189.541
					0.4, 0.04	24	60	18	38	4	1	175.662

**Calculation did not complete*

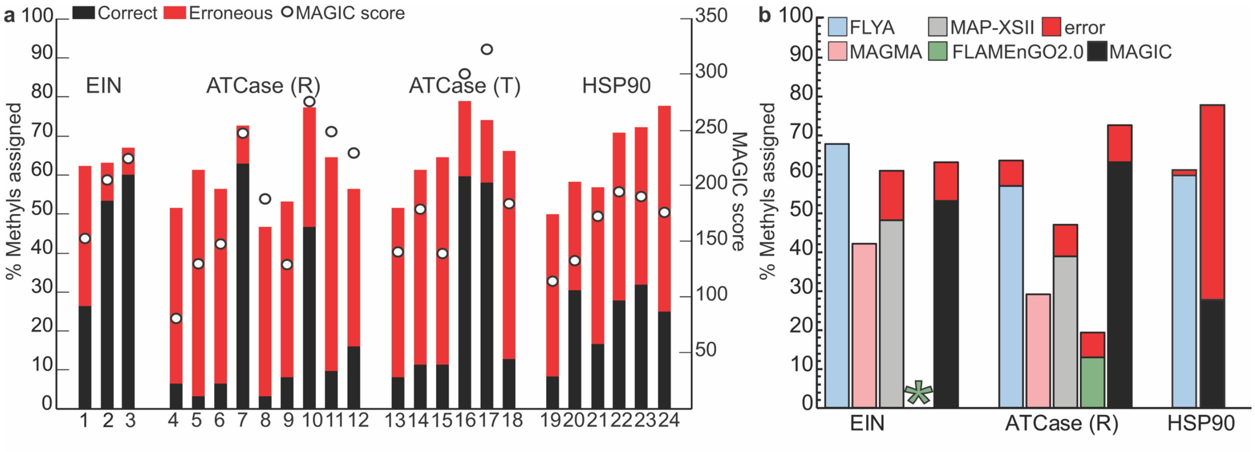

Supplementary Fig. 10 Performance of the MAGIC protocol[4] as summarized in Supplementary Table 6. **a** The x-axis corresponds to the calculation IDs listed in Supplementary Table 6 (column *Calc. ID*). Each calculation ID is associated with a set of parameters for the lower and upper distance thresholds and ^{1}H, ^{13}C peak matching tolerances. The MAGIC score associated with every result is indicated by open circles (scale on the right). **b** Comparison of performance of all protocols on two benchmark proteins, EIN and ATCase and, in addition, for FLYA and MAGIC on HSP90. The parameters that resulted in the highest MAGIC score (A, MAGIC score axis) for the filtered peak lists of EIN and ATCase were chosen (Calc. IDs 2 and 7, respectively). All protocols were compared against the same input peak lists and the same input structure, i.e. the R state structure, PDB ID 1D09, for ATCase. MAGIC and MethylFLYA are additionally compared against the HSP90 data (Supplementary Table 6, Calc. ID 22).

-

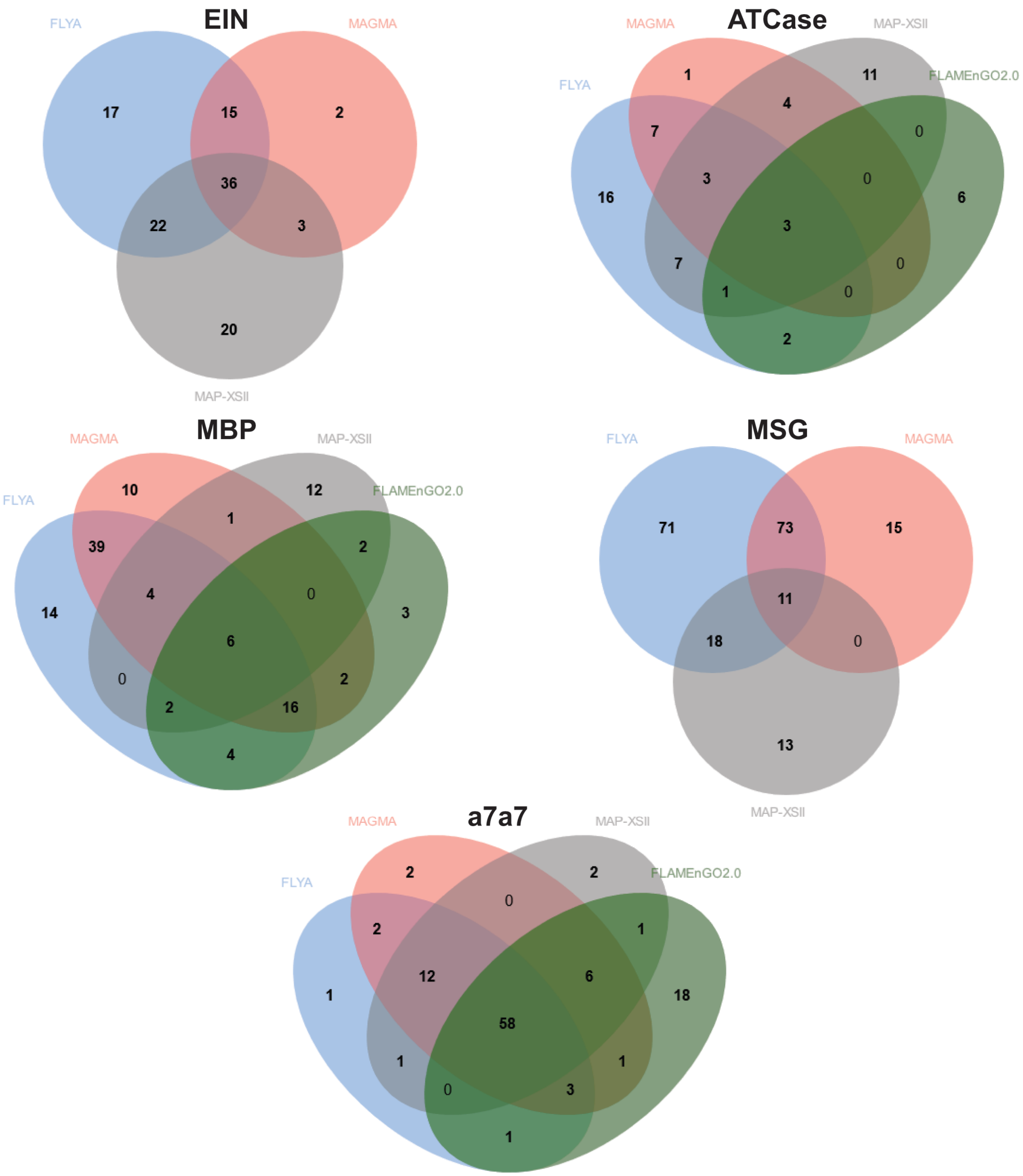

Supplementary Fig. 11 Intersection of assignments generated with different automatic methyl assignment protocols. The illustration of the intersections of assignment solutions from MethylFLYA, MAGMA[1], FLAMEnGO2.0[3], and MAP-XSII[2] are shown for the indicated benchmark cases. In the case of FLAMEnGO2.0, no confident (100%) assignments were found for EIN and MSG.

Supplementary Methods

An example MethylFLYA automated methyl assignment calculation for the N-terminal domain of E. coli Enzyme I (EIN) can be downloaded from http://www.cyana.org/methylflya.tgz.

The complete assignment calculation is performed by first running the *RUN.cya* macro that calls *PREP.cya* to make expected peaks using three different NOE distance cutoffs, and starts parallel FLYA automated assignment runs with *CALC.cya* for the different NOE distance cutoffs. After completing the FLYA runs, consensus chemical shifts are obtained with *CONSOL.cya*, which must be run separately.

Input files:

demo.seq	amino acid sequence
demo.pdb	3D structure
C13HSQC.peaks	2D [^{1}H,^{13}C]-HMQC peak list with amino acid type assignments (peaks are assigned to methyl groups of the correct amino acid type but arbitrary residue number)
HCcCH.peaks	short mixing-time 4D CCNOESY for intraresidual Leu/Val connections
CCNOESY.peaks	4D CCNOESY peak list, unassigned
ref.prot	reference chemical shifts (for comparison only)
init.cya	initialization macro
RUN.cya	automated assignment calculation (calls *PREP.cya*, *CALC.cya*)
PREP.cya	prepare peak lists for FLYA
CALC.cya	run FLYA assignment calculation
CONSOL.cya	determine consensus chemical shifts

The preparation macro, *PREP.cya*, performs the following tasks:

1. The [^{1}H,^{13}C]-HMQC peak list, *C13HSQC.peaks*, which is assigned to methyls of the correct amino acid type (and arbitrary residue numbers that are not used), is split into four amino acid type-specific peak lists, C13HSQC_*X*.peaks, with *X* = A, I, L, V.
2. The peaks in the unassigned short mixing-time 4D CCNOESY peak list, *HCcCH.peaks* (formally treated as an HCCH TOCSY-type experiment), which contains intraresidual connections between the two methyl groups of Leu or Val, are assigned to amino acid types (and irrelevant, arbitrary residue numbers) according to the closest [^{1}H,^{13}C]-HMQC peaks, and the peak list is split into two amino acid type-specific peak lists, *HCcCH_L.peaks* and *HCcCH_V.peaks*.
3. The unassigned 4D NOESY peak list, *CCNOESY.peaks*, is treated similarly, and split into 16 amino acid pair type-specific peak lists, *CCNOESY_XY*.peaks, with *X*, *Y* = A, I, L, V.
4. The macro *peaklists.cya* that specifies the generation of expected peaks during the FLYA calculations in *CALC.cya* is written.

The *RUN.cya* macro creates three subdirectories *demo_d4.5*, *demo_d5.0*, and *demo_d5.5* for the three FLYA calculations with different NOE distance cutoffs, d_{cut} = 4.5, 5.0, 5.5 Å. In each of these three directories, the input files are copied, the *PREP.cya* macro is executed, and three jobs of 100 individual FLYA assignment runs each are started with *CALC.cya*. The individual assignment runs are executed in parallel on different processors, if available.

Subsequently, consensus assignments are generated with the *CONSOL.cya* macro that produces the following main output files:

consol.prot	consensus chemical shift lists (in XEASY format)
consol-strong.prot	strong (confident) consensus chemical shifts (in XEASY format)
consol.tab	table of consensus methyl assignments
consol.pdf	plot of consensus methyl assignments

Optionally (not used in this paper), already known, partial methyl assignments can be included in the calculation by specifying their shifts in a chemical shift list file, e.g. *fix.prot*, and adding the line 'shiftassign_fix := fix.prot' to the *CALC.cya* macro.

For more details on specifying the partial assignments, comparing results to the known reference, or other generic FLYA input file requirements, macros, and output files please see: http://www.cyana.org/wiki/index.php/Tutorials.

Experiment definitions in the CYANA library

The CYANA library (*cyana.lib*) contains definitions of the experiments necessary for automatic methyl resonance assignment with MethylFLYA:

```
SPECTRUM C13HSQC C H
 0.980 C:C_A* H:H_A*

SPECTRUM CCNOESY3D C1 C2 H1
 0.900 C1:C_A* H1:H_A* ~4.0 H_A* C2:C_A*
 0.800 C1:C_A* H1:H_A* ~4.5 H_A* C2:C_A*
 0.700 C1:C_A* H1:H_A* ~5.0 H_A* C2:C_A*
 0.600 C1:C_A* H1:H_A* ~5.5 H_A* C2:C_A*
 0.500 C1:C_A* H1:H_A* ~6.0 H_A* C2:C_A*

SPECTRUM CCNOESY H1 H2 C2 C1
 0.900 C1:C_A* H1:H_A* ~4.0 H2:H_A* C2:C_A*
 0.800 C1:C_A* H1:H_A* ~4.5 H2:H_A* C2:C_A*
 0.700 C1:C_A* H1:H_A* ~5.0 H2:H_A* C2:C_A*
 0.600 C1:C_A* H1:H_A* ~5.5 H2:H_A* C2:C_A*
 0.500 C1:C_A* H1:H_A* ~6.0 H2:H_A* C2:C_A*

SPECTRUM HCcCH H1 H2 C2 C1
 1.000 H1:H_ALI C1:C_ALI C_ALI C2:C_ALI H2:H_ALI
```

The header line of an experiment definition starts with the word SPECTRUM and gives the name of the spectrum type and a list of labels that correspond to the nuclei that constitute the direct and indirect dimensions, one for each spectral dimension.

Subsequent rows specify the magnetization transfer pathways for the experiment. The first number denotes the peak observation probability. It is followed by a linear list of atom types that defines a molecular fragment, in which atoms must be of the given types, e.g. H_ALI for aliphatic hydrogens, H_A* for aliphatic or (for MethylFLYA irrelevant) aromatic hydrogens, C_ALI for aliphatic carbons, etc., as defined in the ATOMTYPES section at the beginning of the CYANA residue library. Atoms must be connected to the next atom in the list either by a covalent bond or, in the instances where a tilde followed by a number is given, by an NOE, i.e. a distance shorter than the given cutoff (in Å) in the 3D structure. An expected peak is generated whenever a molecular fragment matches the covalent structure and, in case of NOEs, the 3D protein structure.

The nuclei for which the frequency is measured in the experiment are identified by labels, followed by a colon. There must be as many labels as in the header (SPECTRUM) line, corresponding to the dimensionality of the spectrum.

See http://www.cyana.org/wiki/index.php/Residue_library_file for more details about the CYANA library.

Input peak list format

Input peak lists must contain a header line starting with #SPECTRUM that specifies the spectrum type and the labels, which must match the corresponding entry in the CYANA library, but may be permuted to indicate the order in which data for the spectral dimensions is given in peak list columns. For instance, a 4D NOESY peak list in XEASY format may start as follows:

```
# Number of dimensions 4
#SPECTRUM CCNOESY H1 C1 H2 C2
 872   0.230  19.382   0.655  13.602 1 U   1.000E+02  0.000E+00 e 0 -  -  -  -
 874  -0.834  17.697   0.655  13.602 1 U   1.000E+02  0.000E+00 e 0 -  -  -  -
 883   0.848  21.207   0.390  11.341 1 U   1.000E+02  0.000E+00 e 0 -  -  -  -
 887   0.924  22.805   0.390  11.341 1 U   1.000E+02  0.000E+00 e 0 -  -  -  -
 894   1.376  25.567   0.390  11.341 1 U   1.000E+02  0.000E+00 e 0 -  -  -  -
 901   0.407  21.750   0.746  16.119 1 U   1.000E+02  0.000E+00 e 0 -  -  -  -
```

Following the header, data for each peak is given on one line: peak number, peak position (ppm; 4 real numbers), "1 U", peak volume, volume error (if known), "e 0", and assignment ('–' if unassigned). Assignments, if present, are given in the form *A.r*, where *A* denotes an atom name and *r* a residue number.

Table of consensus chemical shifts

The consolidation of the chemical shift assignments from individual assignment runs into consensus chemical shifts is documented in the MethylFLYA output file *consol.tab*, which contains the final methyl assignment results. The beginning of an example *consol.tab* file is given below.

```
    Atom  Residue      Ref   Shift     Dev  Extent  inside   inref
    QD1   ILE    2            0.739           300.0   100.0     0.0  strong
    CD1   ILE    2           16.246           300.0    99.9     0.0  strong
    QD1   ILE    5            0.843           300.0    37.1     0.0
    CD1   ILE    5           12.874           300.0    62.7     0.0
    QD1   LEU    6   0.755    0.756  -0.001   300.0    99.5   100.0  strong=
    QD2   LEU    6   0.891    0.891   0.000   300.0    96.4    97.0  strong=
    CD1   LEU    6  23.060   23.065  -0.005   300.0    98.3    98.3  strong=
    CD2   LEU    6  25.480   25.557  -0.077   300.0    96.8    97.0  strong=
```

The first three columns in the file list the atom type, residue type, and residue number for each assigned atom. When a reference assignment is known, the value of the known reference chemical shift is listed in the fourth column. The consensus chemical shift (i.e. the MethylFLYA result) is given in the fifth ("Shift") column. When applicable, its deviation from the reference assignment is given in the 'Dev' column in ppm. The 'Extent' column refers to the number of individual assignments runs, in which an assignment for the given atom was obtained. Given that a hundred calculations are run at each of three distance cutoffs, the consolidation runs over 300 individual calculations. The next two columns 'inside' and 'inref' respectively specify the percentage of

assignments for this atom in the (300) individual runs that agree (within the chemical shift tolerance specified in the init.cya file) with the consensus or reference assignment, respectively. The final column indicates whether an assignment is ‘strong’ (i.e. confident). For the calculations in this paper, assignments are classified as strong if and only if the percentage in the ‘inside’ column is 80% or more. When a reference assignment is known, a ‘=’ is appended to the last column if the MethylFLYA assignment match the reference assignment (within the chemical shift tolerance), or ‘!’ if the two assignments differ by more than the chemical shift tolerance.

Supplementary References

1. Pritisanac, I. et al. Automatic assignment of methyl-NMR spectra of supramolecular machines using graph theory. J. Am. Chem. Soc. 139, 9523–9533 (2017).
2. Xu, Y. Q. & Matthews, S. MAP-XSII: an improved program for the automatic assignment of methyl resonances in large proteins. J. Biomol. NMR 55, 179–187 (2013).
3. Chao, F. A., Kim, J. G., Xia, Y. L., Milligan, M., Rowe, N. & Veglia, G. FLAMEnGO 2.0: An enhanced fuzzy logic algorithm for structure-based assignment of methyl group resonances. J. Magn. Reson. 245, 17–23 (2014).
4. Monneau, Y. R. et al. Automatic methyl assignment in large proteins by the MAGIC algorithm. J. Biomol. NMR 69, 215-227 (2017).
5. Goddard, T. D. & Kneller, D. G. Sparky 3. University of California (2001)
6. Venditti, V., Fawzi, N. L. & Clore, G. M. Automated sequence- and stereo-specific assignment of methyl-labeled proteins by paramagnetic relaxation and methyl-methyl nuclear overhauser enhancement spectroscopy. J. Biomol. NMR 51, 319–328 (2011).
7. Velyvis, A., Schachman, H. K. & Kay, L. E. Assignment of Ile, Leu, and Val methyl correlations in supra-molecular systems: An application to aspartate transcarbamoylase. J. Am. Chem. Soc. 131, 16534-16543 (2009).
8. Shah, D. M., Ab, E., Diercks, T., Hass, M. A. S., van Nuland, N. A. J. & Siegal, G. Rapid protein-ligand costructures from sparse NOE data. J. Med. Chem. 55, 10786–10790 (2012).
9. Würz, J. M. & Güntert, P. Peak picking multidimensional NMR spectra with the contour geometry based algorithm CYPICK. J. Biomol. NMR 67, 63–76 (2017).
10. Han, B., Liu, Y. F., Ginzinger, S. W. & Wishart, D. S. SHIFTX2: significantly improved protein chemical shift prediction. J. Biomol. NMR 50, 43–57 (2011).
